# Transposable elements acquire time- and sex-specific transcriptional and epigenetic signatures along mouse fetal gonad development

**DOI:** 10.3389/fcell.2023.1327410

**Published:** 2024-01-12

**Authors:** Isabelle Stévant, Nitzan Gonen, Francis Poulat

**Affiliations:** ^1^ The Mina and Everard Goodman Faculty of Life Sciences and the Institute of Nanotechnology and Advanced Materials, Bar-Ilan University, Ramat Gan, Israel; ^2^ Institute of Human Genetics, CNRS UMR9002 University of Montpellier, Montpellier, France

**Keywords:** transposable elements, sex determination, gonads, testis, ovary, gene expression regulation

## Abstract

Gonadal sex determination in mice is a complex and dynamic process, which is crucial for the development of functional reproductive organs. The expression of genes involved in this process is regulated by a variety of genetic and epigenetic mechanisms. Recently, there has been increasing evidence that transposable elements (TEs), which are a class of mobile genetic elements, play a significant role in regulating gene expression during embryogenesis and organ development. In this study, we aimed to investigate the involvement of TEs in the regulation of gene expression during mouse embryonic gonadal development. Through bioinformatics analysis, we aimed to identify and characterize specific TEs that operate as regulatory elements for sex-specific genes, as well as their potential mechanisms of regulation. We identified TE loci expressed in a time- and sex-specific manner along fetal gonad development that correlate positively and negatively with nearby gene expression, suggesting that their expression is integrated to the gonadal regulatory network. Moreover, chromatin accessibility and histone post-transcriptional modification analyses in differentiating supporting cells revealed that TEs are acquiring a sex-specific signature for promoter-, enhancer-, and silencer-like elements, with some of them being proximal to critical sex-determining genes. Altogether, our study introduces TEs as the new potential players in the gene regulatory network that controls gonadal development in mammals.

## Introduction

Sex determination is the developmental process by which individuals acquire the necessary organs for sexual reproduction. In vertebrates, it starts with the differentiation of the bipotential gonadal primordium into the ovaries or testes. In mice, the bipotential gonad forms around embryonic day (E) 10.0 on the ventral surface of the intermediate mesoderm and comprises multipotent somatic cells and primordial germ cells (Mayere et al., 2022; Neirijnck et al., 2023). At this stage, the XX and XY somatic cells display no autosomal sexual dimorphism at the transcriptional (Stevant et al., 2019) and chromatin levels (Garcia-Moreno et al., 2019). The supporting cell lineage is the first to operate sex fate decision at E11.5 by differentiating as either Sertoli cells or pre-granulosa cells depending on the presence or absence of the Y chromosome (Albrecht and Eicher, 2001; Chassot et al., 2008). Once specified, Sertoli and pre-granulosa cells instruct other somatic progenitors and the primordial germ cell to commit and differentiate toward the testicular or ovarian cell fates. In XX gonads, the canonical Wnt pathway (WNT4/RSPO1/β-catenin) and the transcription factors (TFs), FOXL2 and RUNX1, induce the differentiation of pre-granulosa cells (Greenfield, 2021). In XY gonads, sex determination is governed by the *Sry* gene located on the Y chromosome (Sinclair et al., 1990; Koopman et al., 1991) that activates the expression of the transcription factor SOX9 at E11.5 (Gonen et al., 2018), which in turn controls the differentiation of Sertoli cells (Vining et al., 2021). In both sexes, the action of master transcription factors induces the activity of sex-specific regulatory networks, controlling the expression and repression of downstream pro-testicular or pro-ovarian genes. Many levels of regulation are expected to play a role in controlling this delicate gene regulatory network. Indeed, it has been shown that prior to sex determination, sex-specific genes carry a bivalent histone mark signature with both active (H3K4me3) and repressive (H3K27me3) markers. As sex differentiation progresses, these genes lose one of these markers and acquire sex-specific expression (Garcia-Moreno et al., 2019). Furthermore, many sex-specific intergenic loci acquire the deposition of H3K27Ac, which characterizes active enhancers (Garcia-Moreno et al., 2019). With two alternative outcomes, the study of sex determination provides an attractive model for studying the epigenetic events involved in cell fate decisions.

We have previously shown that the versatile nuclear-scaffold protein TRIM28 directly interacts and co-localizes with SOX9 on the chromatin of fetal Sertoli cells (Rahmoun et al., 2017). We also discovered that TRIM28 protects adult ovaries from granulosa to Sertoli cell reprogramming through its SUMO E3 ligase activity (Rossitto et al., 2022). Interestingly, in addition to its role on gene regulation as a transcriptional activator or repressor, TRIM28 is a master regulator of transposable element (TE) silencing in somatic cells (Rowe et al., 2010). In mammalian genomes, nearly half of the DNA consists of TEs. TEs are divided into two classes depending on the mechanism by which they transpose. Class I TEs are retrotransposons that propagate via a “copy–paste” mechanism by using an intermediate RNA molecule that is reverse-transcribed as DNA before reinsertion into the host genome. Class II TEs are DNA transposons propagating via a “cut and paste” mechanism. They encode a transposase that excises their flanking inverted terminal repeats and inserts them somewhere else in the host genome. The repression of somatic or germinal TE expression is crucial to block the genetic instability that their uncontrolled expression would induce (Hedges and Deininger, 2007). Therefore, most TE loci are silenced by H3K9me3 or CpG methylation in somatic cells and by the piRNA machinery in male germinal lineages (Zhou et al., 2022). In somatic cells, TRIM28 negatively regulates retrotransposons upon its interaction with KRAB-containing zinc-finger proteins. Consequently, TRIM28 recruits the histone methylase SETDB1 that deposits the heterochromatin mark H3K9me3, contributing to epigenetic retrotransposon silencing (Randolph et al., 2022).

Apart from the potential genomic stability threat, some TEs have become an intrinsic part of the genome during vertebrate evolution and are drivers of genetic innovations, notably in sex determination and reproduction (Dechaud et al., 2019). For instance, TE insertion in sablefish and medaka generated allelic diversification, leading to the creation of a new master sex-determining gene (Schartl et al., 2018; Herpin et al., 2021). More recently, a second exon of the *Sry* gene was identified in mice. This cryptic exon originates from the insertion of four retrotransposons. While the short SRY isoform contains a C-terminal degron, which renders the protein unstable, the long isoform containing the cryptic exon is degron-free and, thus, more stable (Miyawaki et al., 2020). Furthermore, co-option and domestication have repurposed TEs for the benefit of their host and contributed to novel regulatory mechanisms. We distinguish three different mechanisms by which TEs influence gene expression. First, TEs are involved in chromatin organization. TEs containing CCCTC-binding factor (CTCF) motifs were found to be directly involved in the formation of topologically associating domains (TADs) and long-range enhancer–promoter interactions (Lawson et al., 2023). Second, TE sequences are rich in motifs for lineage-specific transcription factors and can be co-opted as *cis*-regulatory elements. For example, endogenous retroviruses (ERVs) were shown to be highly enriched in species-specific placenta development enhancers (Chuong et al., 2013). Finally, TE-derived transcripts were shown to influence gene expression by mechanisms that are still poorly understood. In mice, the transition from the two-cell stage and development progression to the blastocyst stage appeared to depend on LINE-1 expression (Jachowicz et al., 2017; Percharde et al., 2018). TE expression was also shown to be involved in adult neurogenesis, neuronal pathologies, and cancer (Richardson et al., 2014; Jonsson et al., 2020; Jansz and Faulkner, 2021).

In this work, we aimed to characterize TE expression and chromatin landscape as fetal gonads specify as testis or ovary in order to establish the groundwork for further functional studies of TEs in mouse gonadal development. We first identified that common TEs were dysregulated in adult ovaries of *Trim28* KO mice (Rossitto, et al., 2022) and ovarian *Dmrt1* overexpression mice (Lindeman et al., 2015), two models presenting granulosa to Sertoli transdifferentiation, suggesting a sex-specific signature of TE expression in mice. Hence, we reanalyzed transcriptomic and epigenomic data from embryonic gonads and found that the major expression of TE loci is indeed detectable in both sexes with temporal- and sex-dependent variations. We observed that a significant proportion of open chromatin regions contain TE sequences associated to active or repressive histone marks and having enrichment for DNA motifs recognized by transcription factors involved in sex determination. Therefore, our findings suggest that TEs may play a role at several levels during mammalian sex determination.

## Results

### Quantification of TE expression in mouse developing gonads

We first reanalyzed bulk RNA-seq data from control and mutant adult whole ovaries (7 months old), displaying granulosa to Sertoli cell transdifferentiation upon conditional *Trim28* deletion in the granulosa cells (Rossitto, et al., 2022), and measured the change in TE expression. Due to their highly repetitive nature and low RNA abundance, the evaluation of TE expression requires the use of dedicated tools. TE-specific mapping software re-attributes the sequencing reads, habitually discarded from regular RNA-seq, to the TE family or TE loci, allowing the quantification of their expression levels (Lanciano and Cristofari, 2020). For that aim, we used the SQuIRE suite of tools which is based on an expectation–maximization (EM) algorithm to map RNA-seq data and quantify individual TE copy expression (Yang et al., 2019). We found that 8,110 TEs were upregulated upon cell reprograming, while 2,102 were downregulated (Supplementary Figure S1A). We predicted that TE dysregulation is mainly driven by the absence of the TE master regulator TRIM28, but we hypothesized that TEs could also be dysregulated by the change of cell identity. To verify this hypothesis, we also reanalyzed RNA-seq data stemming from the reprograming of adult granulosa to Sertoli cells upon *Dmrt1* forced expression (Lindeman, et al., 2015). While DMRT1 has no identified role in TE expression regulation, we found that 4,099 TEs were differentially expressed upon cell reprogramming (Supplementary Figure S1B). We compared the differentially expressed TEs in the *Trim28* knockout and the *Dmrt1*-induced cell reprograming and found 935 TEs that are commonly dysregulated (Supplementary Figure S1C), supporting the idea of a sex-specific signature of TE expression in the mouse gonads.

**FIGURE 1:**
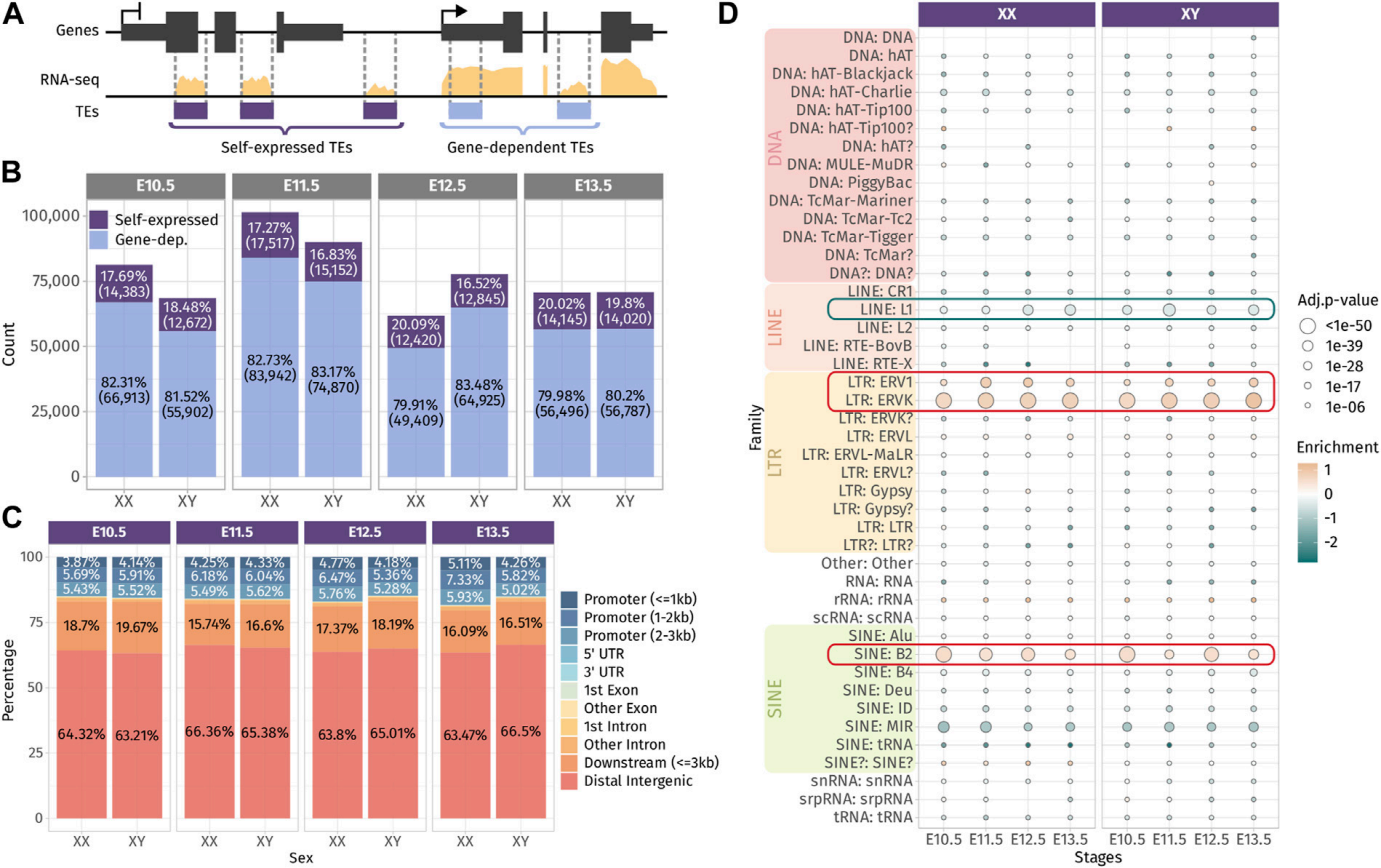
Expressed TEs in the developing gonads. **(A)** Classification of TEs as “self-expressed” when they are found in intergenic regions or within a gene that is not expressed or as “gene-dependent” when they are found within a transcribed gene. **(B)** Number of self-expressed and gene-dependent TEs per sex and stage. **(C)** Repartition of the self-expressed TEs in the genome for each sex and stage. **(D)** Enrichment test of the self-expressed TE families compared to their distribution in the mouse genome. Positively enriched TE families are colored in orange, while the under-represented families are colored in green. Enrichment score is represented as the log2 (odds ratio) from Fisher’s exact test. The size of the dots reflects the statistical power of their enrichment. Only enrichment with an adj.*p*-value<0.05 are shown.

Next, we sought to evaluate the involvement of TE-derived RNAs in the dynamics of gonadal transcriptomes during sex determination. For that aim, we measured TE RNAs at a locus-specific level in mice using the bulk RNA-seq dataset of whole mouse gonads from E10.5, E11.5, E12.5, and E13.5 of both sexes (Zhao et al., 2018). We were able to detect between 61,000 and 101,000 expressed TE loci across all the embryonic stages and sexes (Supplementary Figure S2A). TEs are interspersed throughout the mouse genome and can be located with genes and as such incorporated within gene transcripts. To discriminate TE transcripts induced by their own promoters from passive TE co-transcription with genes, we classified TEs with respect to their environment, as done by others (Chang et al., 2022). We considered expressed TEs found in intergenic regions or inside genes that are not transcribed as “self-expressed” since their expression is likely to be driven by their own promoters, while TEs located within expressed genes are qualified as “gene-dependent” since their transcripts are likely to be part of their host gene RNAs (Figure 1A). Although TEs are relatively rare in gene bodies (Kapusta et al., 2013), we found that up to 83% of the detected TEs were located with expressed genes. On the other hand, self-expressed TEs constitute approximately 17% of the detected TEs, which represents between 12,672 and 17,517 TE loci that are transcribed independently from genes across stages and sexes (Figure 1B). In the present study, we focused our interest on self-expressed TEs as part of autonomously transcribed RNAs susceptible to participate in the gonadal sex differentiation genetic program. As TE expression has been observed in male germ cells (Liu et al., 2014), we verified if self-expressed TEs detected can be expressed by gonadal somatic cells. To this aim, we reanalyzed the only available bulk RNA-seq data from purified somatic cells that was performed at E11.5 in XX and XY gonads (Miyawaki, et al., 2020). We detected 8,696 and 7,479 common self-expressed TEs between whole gonad and somatic cell in XX and XY, respectively, representing nearly half of the self-expressed TEs we detected in whole gonads at E11.5 for both sexes (Supplementary Figure S2C). These results show that self-expressed TEs we detected in the whole gonads are transcribed by both/either the germ cells and/or the somatic cells of the gonads.

We question whether self-expressed TEs were located in a close proximity of genes, in particular to promoter regions and 3′ end regions, as TEs have been described to produce alternative promoters, transcription start sites (TSSs) and transcription termination sites (TTSs) of genes (Conley and Jordan, 2012; Miao et al., 2020). We found that approximately 15% of self-expressed TEs were located in gene promoter regions up to 3 kb from the gene TSS, and 20% of them were found in the 3-kb downstream of the 3′ end of the genes. The vast majority of self-expressed TEs (65%) were distal intergenic, located more than 3 kb away from genes (Figure 1C). Then, we performed an enrichment analysis to detect the over- or under-represented family of self-expressed TEs compared to the distribution of TE families along the mouse genome (Figure 1D). The enrichment test revealed that DNA families were under-represented among self-expressed TEs, which is expected as they are mostly inactive and only exist as the relics of anciently active elements (Platt et al., 2018). We also noticed that the LINE family, in particular the LINE-1 subfamily, was under-represented. LINE-1 represents 24.4% of the mouse TEs, and ∼1,000 copies are potentially capable of active retrotransposition (Goodier et al., 2001). While LINE-1 is highly expressed in the mouse preimplantation embryos (Jachowicz, et al., 2017), fewer copies than expected (8% less) were found transcriptionally active in the developing gonads (Figure 1D and Supplementary Figure S2C). Conversely, we observed a positive enrichment for ERV1 and ERVK LTRs, as well as the B2 SINE non-autonomous retrotransposons.

Overall, we found that TEs are broadly expressed along gonadal development and a large proportion of the detected TEs are a constituent part of genes. The detected self-expressed TE loci from the whole gonads are potentially transcribed in both the somatic and germ cell compartment of the gonads. Self-expressed TEs are mostly found in distal intergenic regions and are enriched in ERVs and SINE B2 retrotransposon families.

### Mouse gonads exhibit the gradual sex-specific expression of TEs

**FIGURE 2:**
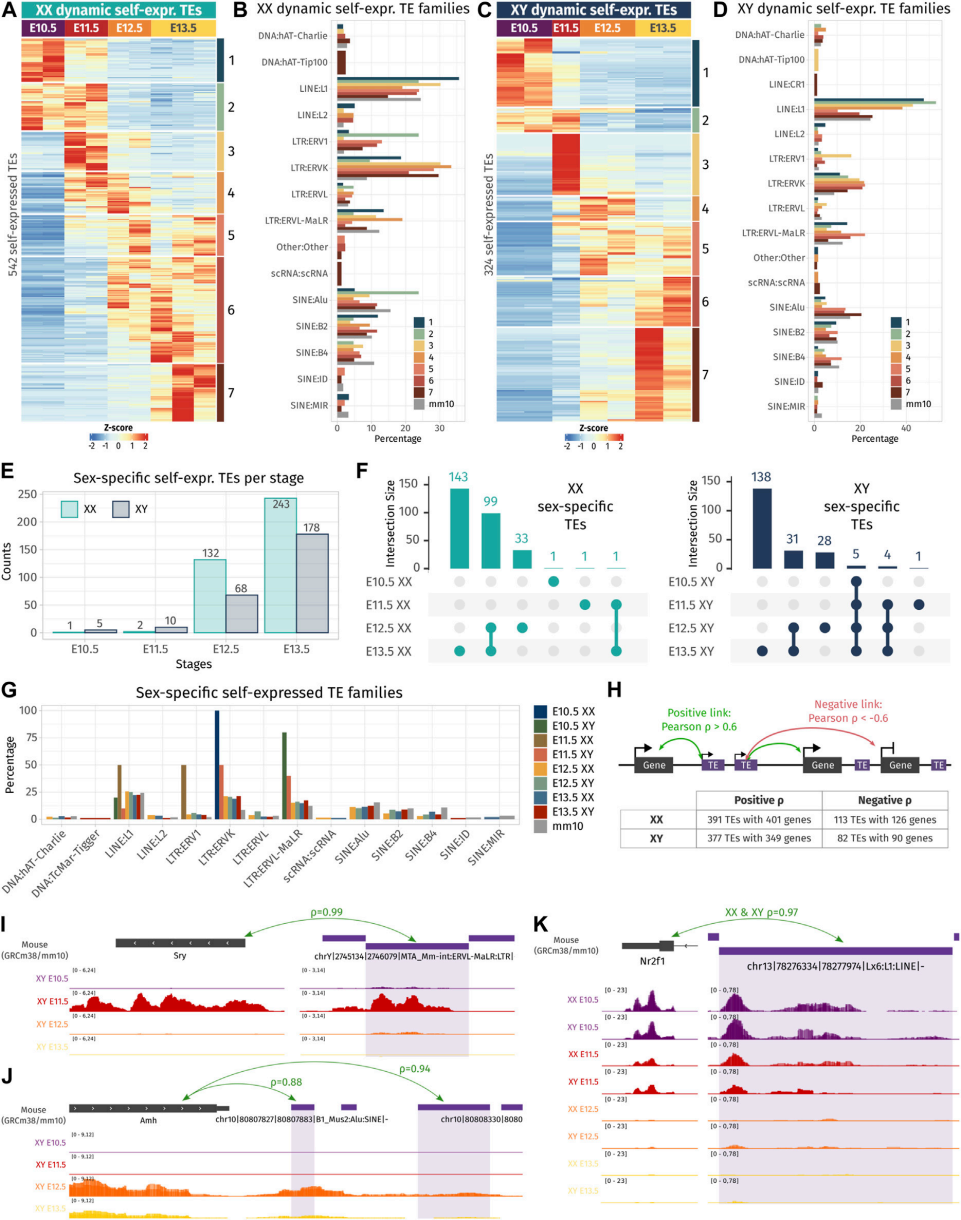
Dynamic- and sex-specific TEs along gonadal development. **(A, C)** Heatmap showing the self-expressed TEs that are differentially expressed among different embryonic stages in XX **(A)** and XY **(C)** gonads. The expression is normalized by a z-score. **(B, D)** Percentage of TE families for each cluster of an expression pattern. As reference, the global percentage of TE families in the mouse genome is shown in gray. **(E)** Number of overexpressed self-expressed TEs between sexes for each stage. **(F)** Upset plots representing the overlap of the sex-specific TEs across embryonic stages. The bars on the top represent the number of TEs found in each intersection. The dots linked by lines represent the intersections. **(G)** Percentage of TE families of the sex-specific TEs found at each embryonic stage. **(H)** Schematic representation describing the strategy to find the correlation between TEs and their nearby genes. A TE is correlated to a gene in its 100 kb flanking region if the correlation coefficient is above 0.6 (positive correlation) or below −0.6 (negative correlation). The table shows the number of correlating TE and genes found by the analysis. **(I–K)** Genomic tracks of the RNA-seq signal of three TE-gene positive correlation examples.

After having depicted the TE expression landscape in developing gonads, we undertook to identify if the self-expressed TE loci change in expression during gonadal differentiation and if they exhibit sexual dimorphism. First, we performed differential expression analysis across embryonic stages in both sexes separately, and second, between XX and XY gonads for each stage. Regarding TE expression throughout gonadal development, we identified 542 and 324 TEs presenting a dynamic expression along gonadal specification as ovary or testis, respectively, with 124 of them being shared between the two sexes (Figures 2A–D; Supplementary Data S1). Dynamically expressed TEs were classified in seven groups (numbered 1–7) according to their expression profile. We observed that TEs are expressed in successive waves along gonadal development, with some TEs overexpressed at only one stage (groups 1, 3, and 7 in XX and XY), while other TEs overexpressed during several stages (groups 2, 4 to 6 in XX and groups 2 and 5 XY gonads). In XX gonads, we discovered that LTR:ERV1 and ERVK were more represented than expected among different groups of dynamically expressed TEs (Figure 2B), while in XY gonads, only the LTR: ERVK family was more represented than expected in all dynamically expressed TE groups (Figure 2D). We also discovered that TEs specifically overexpressed at early stages (E10.5 and E11.5) are mainly from the LINE: L1 family in both sexes.

Concerning sexual dimorphism, E10.5 and E11.5 gonads showed few sexual dimorphisms in terms of TE expression (Figure 2E). At E10.5, only one self-expressed TE located in chromosome 6 was found overexpressed in XX compared to XY gonads, and five were found overexpressed in XY compared to XX gonads. These five TEs are located on the Y chromosome and are expressed in all the stages (Figure 2F and Supplementary Data S2). At E11.5, two self-expressed TEs were overexpressed in XX gonads, and ten were found overexpressed in XY, including six TEs located on the Y chromosome stages (Figure 2F and Supplementary Data S2). These results show that autosomal TEs are not expressed in a sexually dimorphic manner in gonadal cells prior to sex determination. From E12.5, we observed an increase of sexually dimorphic self-expressed TE loci, with 200 and 421 loci differentially expressed between XX and XY gonads at E12.5 and E13.5, respectively. This progression of TE expression perfectly copies what was previously observed, concerning the dynamics of the expression of the protein-coding genes (Nef et al., 2005; Jameson et al., 2012; Zhao, et al., 2018; Stevant, et al., 2019). Finally, we looked at whether a particular TE family could be expressed in a sexually dimorphic manner, and no drastic difference was observed compared to the normal proportion of TE families in the mouse genome (Figure 2G).

As TE expression can influence the expression level of their nearby genes, we sought to investigate if the expression level of the identified differentially expressed TEs both in time and sex correlates with proximal gene expression. Positive TE-gene correlation can happen for several reasons. The TE transcript can influence the expression of the gene as a non-coding RNA or enhancer RNA (Liang et al., 2023), TE can act as a promoter or an alternative 3′ UTR for the gene, the gene can be silenced as a collateral effect of the TE silencing, or the TE expression is activated by the same *cis* or *trans* regulatory elements as the gene. On the contrary, negative TE–gene correlation can suggest a negative control of the nearby gene by the TE transcript. As most self-expressed TEs were found in a window of 100 kb of a gene TSS (Supplementary Figure S2D), we looked at the genes present in a 100-kb window around each differentially expressed TE in time and sex, and we calculated Pearson’s correlation between the gene and TE expression among embryonic stages and sexes (Figure 2H). In XX gonads, 391 TE loci were positively correlated with 401 genes, and 113 TEs were negatively correlated with 126 genes. In XY gonads, 377 TEs were positively correlated with 349 genes, while 82 TEs negatively correlated with 90 genes (Supplementary Data S3). Interestingly, among them, we found an LTR ERVL-MaLR retrotransposon located 81 kb upstream of *Sry* expressed with an extremely strong correlation with *Sry* gene expression (ρ = 0.99, Figure 2I). This ERVL-MaLR is separated from *Sry* by one unexpressed gene (*H2al2b*), demonstrating that its expression was produced by a distinct transcript, conversely to the recently identified *Sry* cryptic exon (Miyawaki, et al., 2020). Interestingly, the expression of this TE was also retrieved in RNA-seq data from XY somatic cells purified at E11.5 (Miyawaki, et al., 2020), which strongly suggests that it is expressed in the same cell lineage as *Sry*. We also found a SINE B4 retrotransposon located 2.9 kb downstream of the *Amh* gene with a correlation coefficient of 0.94 (Figure 2J). This specific transposon was not expressed in XX gonads where *Amh* is silenced. Its proximity with the *Amh* 3′ UTR and the presence of other expressed TEs in the vicinity can suggest that the transcript SINE B4 may be part of an alternative 3′ UTR. Finally, we found five LINE-1 TEs positively correlating with the *Nr2f1* gene (Figure 2K and Supplementary Data S3), also known as *Coup-TFI*, which is expressed in the gonadal somatic cells prior to sex determination in both sexes (Stevant, et al., 2019). *Nr2f1* is a pleiotropic gene capable of activating and repressing target gene expression by interacting with chromatin remodelers. It has been described as a driver of cell fate decision in mouse and human brain development (Bertacchi et al., 2019). The expression of *Nr2f1* has been shown to be regulated by the non-long coding RNA *lnc-Nr2f1* (also referred to as A830082K12Rik), which is located directly upstream of Nr2f1 on the opposite strand and is conserved between mice (Bergeron et al., 2016) and humans (Ang et al., 2019). The five expressed TEs correlating with *Nr2f1* expressed are located in the intronic region of the longest version of *lnc-Nr2f1* (Ensembl mm10 annotation). As *Nr2f1* is expressed only in early gonads prior to gonadal cell differentiation, we speculate that it plays a role in the establishment of the bipotential progenitor cell identity.

To summarize, we found that self-expressed TEs are dynamically expressed and acquire sexual dimorphism during gonadal differentiation. Dynamic and sexually dimorphic TE expression correlates with nearby genes, some of which have a pivotal role in gonadal development. Although we cannot determine with the present data whether TE expression precedes their nearby gene transcription, this suggests that TEs are a whole part of the genetic program driving gonadal specification.

### TE loci represent one-third of open chromatin regions in embryonic supporting cells

**FIGURE 3:**
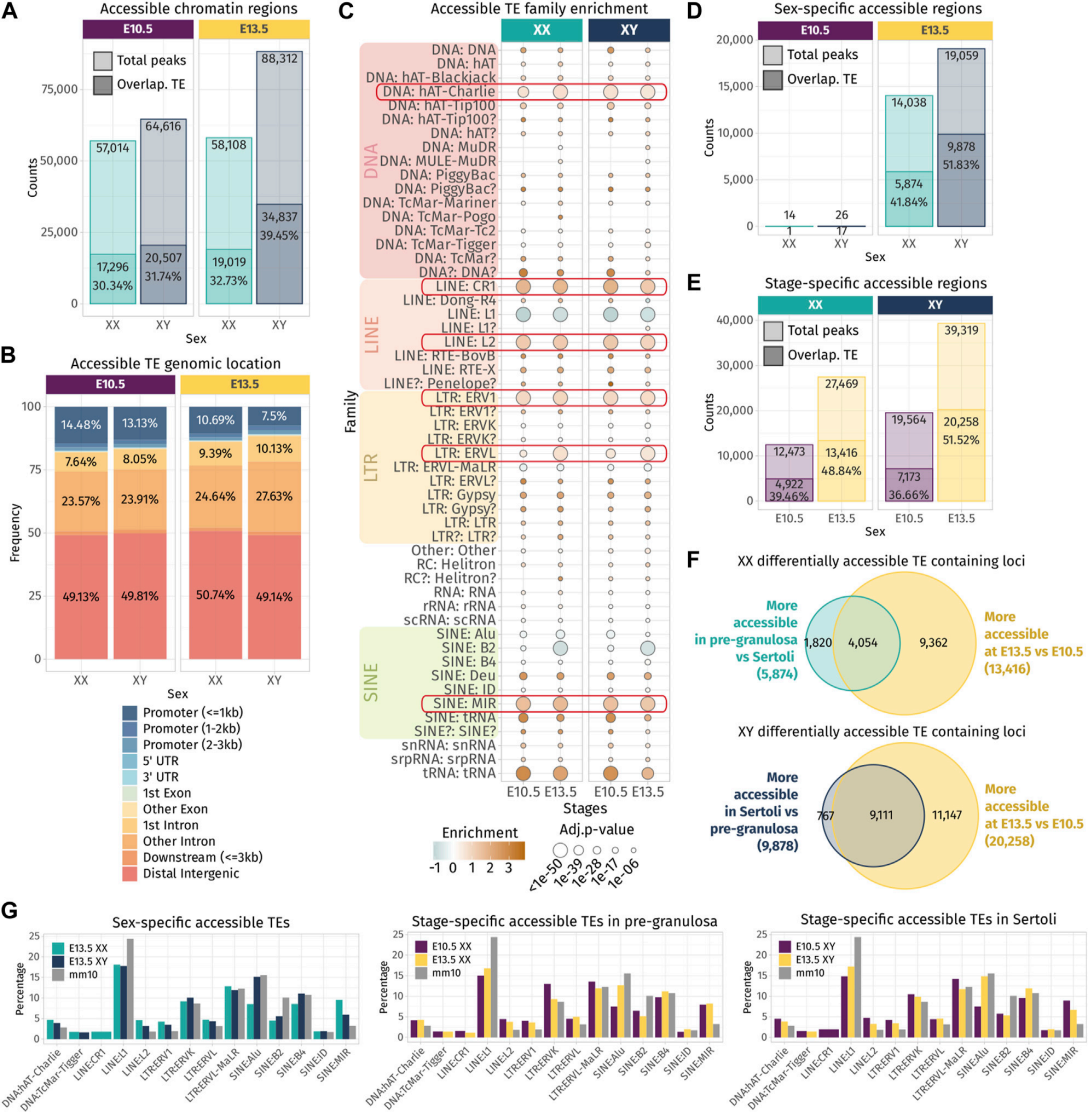
Chromatin accessibility landscape of TE loci during gonadal development. **(A)** Number of ATAC-seq peaks detected in total and overlapping TE loci at E10.5 and E13.5 in XX and XY gonadal progenitor and supporting cells. **(B)** Genomic location of the accessible chromatin regions that overlap with a TE sequence. **(C)** Family enrichment analysis of the TE overlapping ATAC-seq peaks. Positively enriched TE families are colored in orange, while the under-represented families are colored in green. Enrichment score is represented as the log2 (odds ratio) from Fisher’s exact test. The size of the dots reflects the statistical power of their enrichment. Only enrichment with an adj.*p*-value<0.05 is shown. **(D, E)** Statistically differentially accessible regions in sex and time. The number and the percentage of accessible regions and TE-containing loci showing an increase in accessibility between sex and time are indicated. **(F)** Overlapping of the accessible TE loci that increase in accessibility between sex at E13.5 in either Sertoli and pre-granulosa cells (turquoise and dark blue) with the accessible TE loci that gain accessibility at E13.5 compared to E10.5 (yellow). **(G)** Percentage of TE families in the differentially accessible chromatin regions that overlap with TE sequences (only percentages >1 are shown).

Independent of their transcription, TE sequences can influence gene expression by acting as *cis*-regulatory elements including promoters or enhancers by providing a large repository of potential transcription factor-binding sites. A comparative study in mouse developing tissues showed that 21% of the open chromatin regions were associated with TEs and half of them were tissue-specific, suggesting an active role in mouse organogenesis (Miao, et al., 2020). We questioned whether the chromatin around TE loci is also specifically opened, while the bipotential gonads differentiate as ovary or testis. We took advantage of the pioneer epigenetic investigations on mouse embryonic gonads performed by Garcia-Moreno et al. (2019) and Garcia-Moreno et al. (2019), and the ATAC-seq data of purified gonadal somatic cells at E10.5 and supporting cells at E13.5 in both sexes were reanalyzed to identify nucleosome-depleted TEs. In total, we identified between 57,014 and 88,312 accessible chromatin regions. Despite inherent mappability issues due to the TE repetitive sequence nature (i.e., multi-mapped reads are filtered out from the ATAC-seq data), we found that 30%–39% of the ATAC-seq peaks were overlapping TE loci across sexes and stages (Figure 3A). In most cases, more than half of the length of TE sequences was covered by an ATAC-peak, showing that most TE sequences are accessible for potential regulatory factor binding (Supplementary Figure S3). Nearly half of the accessible TE-containing loci are found in the distal intergenic compartment, in which approximately 35% of the loci are located within introns and 10% are found in promoter regions (Figure 3B).

The enrichment test shows that many TE families were over-represented in the open chromatin regions compared to their global proportion in the genome (Figure 3C), including the DNA hAT-Charlie, the LINE CR1 and L2, the LTR ERV1, ERVL, and the SINE MIR. These six TE families were also found enriched in TE-derived enhancer-like sequences identified in different human tissue cell lines (Cao et al., 2019), suggesting that they are not necessarily specific to our model and that some TE families are more potent to contribute as *cis*-regulatory elements than others across mammals. We also noticed that the SINE B2 family, which was enriched among the transcribed TE loci in the whole developing gonad (Figure 1D), is under-represented among the open chromatin regions in supporting cells for both sexes. As we assume transcribed TE loci to be accessible, we can speculate that the B2 family is not preferentially transcribed in the supporting cells.

Next, we performed differential chromatin accessibility analysis between sexes and stages in order to identify the regions that gain in accessibility while gonadal progenitor cells differentiate as either pre-granulosa or Sertoli cells. As previously reported, almost no differences were found in open chromatin regions between XX and XY at E10.5 when the cells are multipotent (Garcia-Moreno, Futtner, et al., 2019). In contrast, at E13.5, we observed 14,038 genomic regions with increased accessibility in pre-granulosa cells compared to Sertoli, and 41.84% of them were overlapping with TE loci. On the other hand, 19,059 genomic regions showed increased accessibility in Sertoli cells compared to pre-granulosa, and 51.83% of them contained TE loci (Figure 3D). We also looked at the genomic regions that increased in accessibility in a time-specific manner in both sexes and found that 27,469 and 39,319 regions were more accessible at E13.5 in pre-granulosa and Sertoli cells, respectively, compared to the E10.5 progenitor cells (Figure 3E). Among them, 48.84% and 51.52% were overlapping TE loci in pre-granulosa and Sertoli cells, respectively. Finally, we found that 30% and 45% of TE loci increasing in accessibility at E13.5 in pre-granulosa and Sertoli cells, respectively, were also sexually dimorphic (Figure 3F). Finally, we investigated whether a particular TE family could be enriched in a sex- or time-specific manner, but we observed no noticeable differences in their proportion among the differentially accessible TE-containing loci, apart for the SINE:Alu elements, which are more prevalent in the Sertoli-specific open loci compared to pre-granulosa, in the pre-granulosa compared to E10.5 XX progenitor cells, and in the Sertoli compared to XY progenitor cells (Figure 3G).

Altogether, these results show that TE loci are increasing in accessibility in a time- and sex-specific manner as supporting cells differentiate as pre-granulosa or Sertoli cells. The fact that more than half of the accessible TEs are located in the intergenic region suggests they could participate in the acquisition of the supporting cell-type identity as active *cis*-regulatory regions.

### TEs acquire the sex-specific enhancer- and promoter-like chromatin landscape as supporting cells differentiate

**FIGURE 4:**
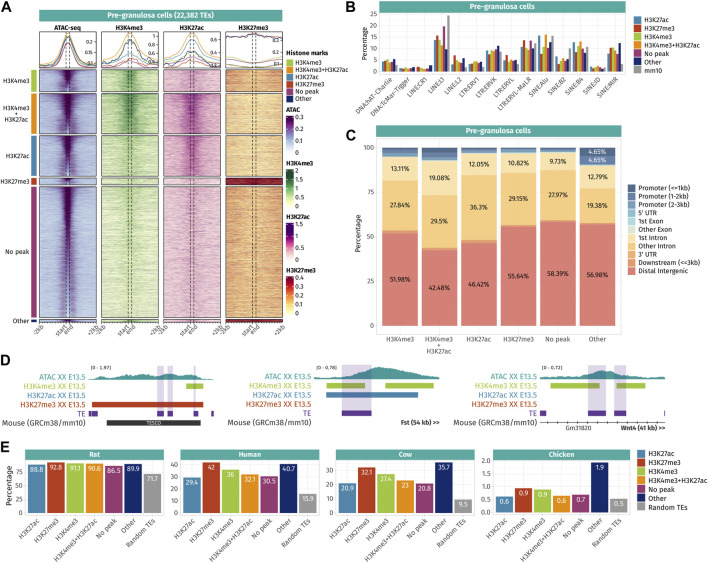
Epigenetic landscape of pre-granulosa cell-accessible TE loci. **(A)** Density plots and heatmaps representing the ATAC-seq and the ChIP-seq signals for histone marks H3K4me3, H3K27ac, and H3K27me3 around the TE loci that gained accessibility in a sex- or time-specific manner in pre-granulosa cells. Accessible TEs were grouped according to the histone marks they display. The groups of histone marks with fewer than 500 TEs were grouped in the “Other” section, as the groups were not clearly visible on the final heatmap. **(B)** Percentage of TE families in different groups of histone marks in pre-granulosa cells (only percentages >1 are shown). **(C)** Genomic location for each chromatin landscape group of TE loci that gains accessibility in a sex- or time-specific manner in pre-granulosa cells. **(D)** Genomic tracks showing the ATAC-seq signal and H3k4me3, H3K27ac, and H3K27me3 ChIP-seq peaks on accessible TE showing enhancer-like chromatin markers and that are close to important gonadal genes. **(E)** Percentage of mouse accessible TE loci conserved among rats, humans, cows, and chickens. TEs are classified according to their histone marks. As a reference, we show the percentage of conservation of 50,000 random TE loci (in gray).

**FIGURE 5:**
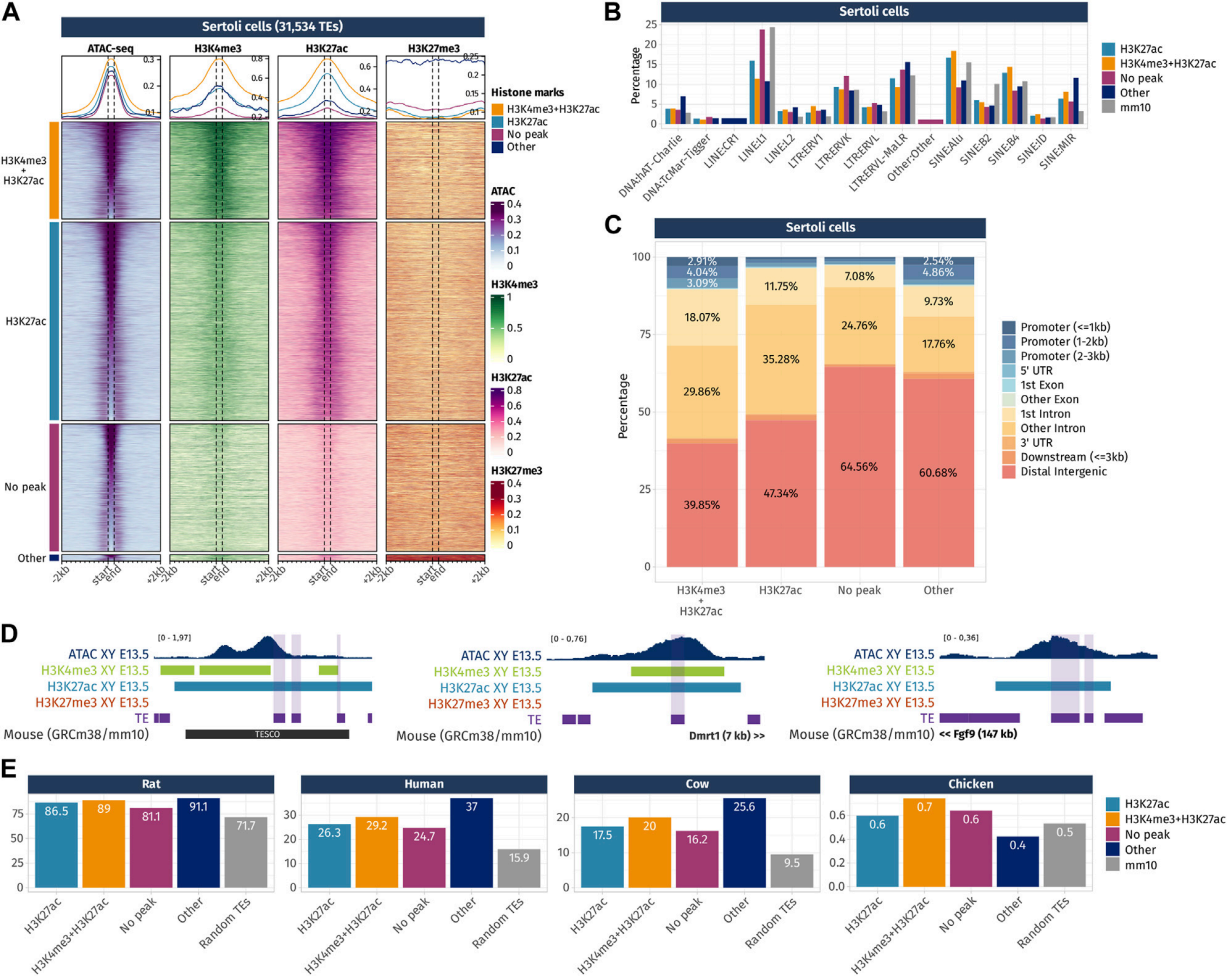
Epigenetic landscape of Sertoli cell-accessible TE loci. **(A)** Density plots and heatmaps representing the ATAC-seq and the ChIP-seq signals for histone marks H3K4me3, H3K27ac, and H3K27me3 around the TE loci that gained accessibility in a sex- or time-specific manner in Sertoli cells. Accessible TEs were grouped according to the histone marks they display. The groups of histone marks with fewer than 500 TEs were grouped in the “Other” section as the groups were not clearly visible on the final heatmap. **(B)** Percentage of TE families in the different groups of histone marks in Sertoli cells (only percentages >1 are shown). **(C)** Genomic location for each chromatin landscape group of the TE loci that gains accessibility in a sex- or time-specific manner in Sertoli cells. **(D)** Genomic tracks showing the ATAC-seq signal and H3k4me3, H3K27ac, and H3K27me3 ChIP-seq peaks on accessible TE showing enhancer-like chromatin markers and that are close to important gonadal genes. **(E)** Percentage of mouse accessible TE loci conserved among rats, humans, cows, and chickens. TEs are classified according to their histone marks. As a reference, we show the percentage of conservation of 50,000 random TE loci (in gray).

To reveal the potential roles of TE sequences that became accessible in pre-granulosa and Sertoli cells in a time- and sex-specific manner, we explored the histone post-transcriptional modifications present in their vicinity. We reanalyzed ChIP-seq data for H3K4me3 (marker of promoters), H3K27ac (marker of active promoters and enhancers), and H3K27me3 (marker of silencers and transcriptionally silenced genes by PRC2) performed on purified gonadal somatic cells at E10.5 and supporting cells at E13.5 in both sexes (Garcia-Moreno et al., 2019; Garcia-Moreno et al., 2019). We classified accessible TEs according to the combination of histone mark peaks overlapping with the TE sequences +/-200 bp to have an overview of the chromatin landscape at a one nucleosome resolution (Supplementary Data S4). Finally, we represented ATAC-seq and the different ChIP-seq signals on accessible TEs and up to 2 kb around them as juxtaposed heatmaps for the pre-granulosa and Sertoli cells (Figures 4A, 5A).

In pre-granulosa cells, TEs were classified into six histone combination groups (Figure 4A), and we determined TE families present in each groups (Figure 4B). The first two groups contain open TEs displaying histone marks specific for promoters (H3K4me3 and H3K4me3 + H3K27ac). They represent 25.6% of the total pre-granulosa-enriched accessible TEs (5,727 out of 22,328 TEs). Surprisingly, only 6.3% of them (360 TEs) were found in a gene promoter region (up to 3 kb upstream of a gene TSS) (Figure 4C), but no biological GO term was statistically enriched among these genes. We examined whether the genes having TEs that increased in accessibility in their promoter showed a concomitant increase in expression. Although RNA-seq was performed in whole gonads and not purified granulosa cells, we found that 55% (181 out of 333 genes) showed an increase in expression between E10.5 and E13.5 [log2 (fold change)>0]. We investigated the closest genes of 5,727 H3K4me3 and H3K4me3 + H3K27ac TEs and found critical pre-granulosa cell markers, such as *Wnt4*, *Fst*, *Runx1*, *Lef1*, and *Kitl* (Figure 4E, Supplementary Data S4). GO term analysis confirmed the statistical enrichments of terms related to reproductive development and Wnt signaling (Supplementary Data S5). The third group of pre-granulosa-enriched accessible TEs displays the H3K27ac histone mark which suggests that they are potential enhancers (3,725 out of 22,382). The proximal genes were enriched for the biological process GO term of sex differentiation and included *Gata4*, *Cited2*, *Kitl*, and *Lhcgr* (Supplementary Data S5). Next, the fourth group contains accessible TEs with the H3K27me3 histone mark that labels silenced genes and silencer elements (638 out of 22,328). Most of them are found in distal intergenic regions (Figure 4C). Interestingly, we found two H3K27me3 TEs (a SINE B4 and a DNA TcMar-Tigger) in close proximity with *Sox9* (Figure 4E)*,* which are located within TESCO (testis-specific enhancer of the *Sox9* core) (Sekido and Lovell-Badge, 2008). We also found a negative regulator of the Wnt pathway, *Sfrp1*, that is expressed in the gonadal progenitor cells but silenced upon the differentiation of the supporting cell lineage of both sexes (Stevant, et al., 2019 17133, Warr et al., 2009). Finally, 53.8% of the pre-granulosa-accessible TEs were not overlapping with any of the investigated histone marks. The proximal genes of these TEs were enriched for female sex differentiation and regulation of canonical Wnt signaling pathway GO terms (Supplementary Data S5). Finally, we investigated whether sex and time differentially accessible TEs were conserved among different species (Figure 4E). We found that independent of their histone marks, the accessible TEs were more conserved than expected among the mammals (rat, human, and cow), but poorly conserved in chickens. Moreover, we observed that most of the accessible TEs were conserved with rats, but to a lesser extent with humans and cows, implying that most of the accessible TEs are rodent-specific.

In Sertoli cells, TEs were classified into four histone combination groups (Figure 5A), and we investigated TE families present in each groups (Figure 5B). The first group is composed of TEs displaying promoter-like histone marks H3K4me3 + H3K27ac (7,124 out of 31,534 TEs). As observed in pre-granulosa, only 10% (715 TEs) of them are found in the promoter region of genes (Figure 5C and Supplementary Data S4). The second group shows TEs with enhancer histone mark H3K27ac (17,570 out of 31,534). Similarly, for the pre-granulosa-enriched accessible TEs, the proximal genes were statistically enriched not only for sex determination-related GO terms but also for terms reflecting Sertoli cell functions, such as epithelium morphogenesis and angiogenesis, among other terms (Supplementary Data S5). Notably, TESCO SINE B4 observed in the pre-granulosa cells and marked with H3K27me3 is present in the H3K27ac Sertoli-accessible TEs (Figure 5D). Although the chromatin state of the present TE is most likely a consequence of its presence within TESCO, the presence of TE loci within a known cell-type-specific enhancer questions on their involvement in the enhancer activity. Interestingly, we found accessible TEs harboring H3K4me3 and H3K27ac histone marks with a 7 kb upstream of *Dmrt1* and 10 kb upstream of *Sox9*. We also found several accessible TEs marked with H3K27ac (Figure 5D), with three of them located 525 kb downstream of *Fgf9* (Supplementary Data S4). These three TEs are contained within the 306 kb locus previously identified as an *Fgf9* enhancer-containing locus using the 3D genome enhancer hub prediction tool. The deletion of the 306-kb region causes partial to complete male-to-female sex reversal (Mota-Gómez et al., 2022). The last group contains TEs showing none of the investigated histone marks (9,367 out of 31,534). Finally, we investigated the accessible TE conservation (Figure 5E) and found that, similar to what was observed in pre-granulosa, TEs were more conserved than expected among the mammals (rat, human, and cow), but poorly conserved in chickens.

Together, these results show that TEs that gain accessibility in a time- and sex-specific manner in pre-granulosa and Sertoli cells upon sex determination are also showing promoter- and enhancer-like properties, which implies they are acting as *cis*-regulatory elements during cell differentiation. The presence of TEs within known critical sex determination enhancer loci such as TESCO or the downstream *Fgf9* enhancer suggest the presence of other TE sequences involved in the process. The absence of either H3K4me3 or H3K27ac on a large fraction of accessible TEs interrogates on the possibility of the presence of other histone marks, such as the recently described H4K16ac that contributes to TE transcription and *cis*-regulatory activity (Pal et al., 2023). Finally, both pre-granulosa and Sertoli cell sex- and time-specific accessible TEs show a higher conservation than expected among mammalian species, suggesting that these specific TEs could have functional significance in mammalian genomes, such as taking part in the genome 3D structure or in cis-regulatory regions.

### Pre-granulosa and Sertoli-accessible TEs are enriched in gonad transcription factor-binding motifs

**FIGURE 6:**
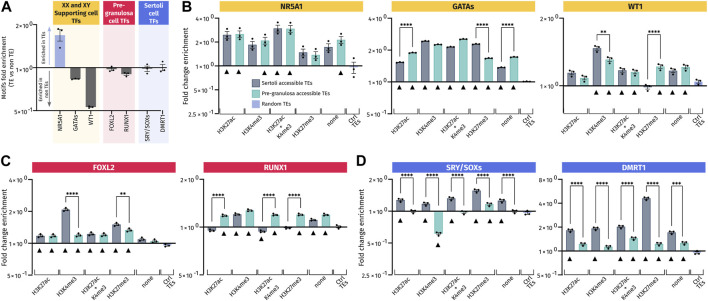
Transcription factor-binding motif enrichment in pre-granulosa- and Sertoli cell-accessible TE loci. **(A)** Ratio of the number of the transcription factor motifs found in three sets of 10,000 randomly picked TE or non-TE sequences. For each set of sequences, motifs analyzed were normalized in matches per kb and were expressed (in Log2) by the ratio of matches per kb in TE/matches per kb in non-TE. **(B–D)** Enrichment for the transcription factor motif in motifs per kb of the different analyzed sets of accessible TEs reported to the motifs per kb of three different sets of 10,000 control TEs. Black triangles mark the accessible TEs having a significant enrichment of each motif compared to control TEs (ctrl TEs) (see details of significance in Supplementary Data S6). Motifs in **(B)** are those recognized by transcription factors involved in the specification of XY and XX somatic progenitor cells. Motifs in **(C)** are those for transcription factors involved in the differentiation of pre-granulosa cells, and motifs in **(D)** are corresponding to Sertoli cells.

TE frequently contains sequences that can attract cell-specific transcription factors to promote their expression and, hence, their transposition. Consequently, TE transposition has dispersed transcription factor-binding sites throughout mammalian genomes over evolution. Numerous studies on TE expression in stem cells and early embryos showed that the active TE families were enriched in binding motifs for pluripotency transcription factors, such as NANOG, OCT4, or SOX2. Furthermore, tissue-specific TE transcription and *cis*-regulatory element activity revealed they contain lineage-specific transcription factor-binding sites (Fueyo et al., 2022). As such, we questioned whether TE loci that gain accessibility in a time- and sex-specific manner in pre-granulosa and Sertoli cells are enriched in motifs for the gonadal transcription factors and, in particular, if the motif enrichment changes depending on the chromatin landscape or the sex. We first carried out *de novo* pattern analyses (Santana-Garcia et al., 2022) that did not provide easily interpretable results due to the low quantitative aspect of this method (Supplementary Table S1). Then, we performed a specific transcription factor motif enrichment analysis for known critical gonadal factors: GATA4/6 (Padua et al., 2014; Padua et al., 2015), NR5A1 (Luo et al., 1994), WT1 (Kreidberg et al., 1993), FOXL2 (Schmidt et al., 2004), RUNX1 (Nicol et al., 2019), SRY/SOX (Sinclair, et al., 1990; Wagner et al., 1994; Portnoi et al., 2018), and DMRT1 (Raymond et al., 2000) (Supplementary Figure S5). We used an approach of motif-scanning that calculates the enrichment of motif occurrence per kb compared to control sequences. We first examined if TE sequences are naturally enriched in gonadal transcription factor motifs. To this aim, we selected 3 × 10,000 random TE loci across the genome and compared their sequence composition to motifs with 3 × 10,000 random DNA regions that are not TEs (Figure 6A). We found that the motif recognized by NR5A1, which is involved throughout gonadal differentiation in mammals, was enriched in randomly selected TE loci (control TEs) from the mouse genome when compared to regions containing no repeated elements (non-TE control). For the other motifs analyzed, we did not find any significant enrichment in TE; some motifs such as GATAs, WT1, and RUNX1 are even under-represented in TEs. However, by analyzing TEs by classes (LINEs, LTRs, SINEs, etc.) for the same datasets, we found that the enrichment of NR5A1 motifs was essentially obtained from SINEs and to a lesser extent from LTRs (Supplementary Figure S4A), while LINEs and others were depleted for this motif. Interestingly, SINEs also display enrichment for FOXL2 and DMRT1 motifs. Our results suggest that if certain motifs pre-exist in TEs, it may be in specific classes of TEs, as we observed here for SINEs.

Then, we compared the accessible TEs harboring or not harboring some histone marks to the control TEs randomly distributed in the genome. All the gonadal motifs examined were enriched in almost all the analyzed TE regions compared to the control TEs, regardless of the associated histone mark (Figures 6B–D). This suggests that TEs recruited during gonadal differentiation may have acquired these motifs *de novo*. It is particularly the case for the NR5A1 motif (Figure 6B) that is already enriched when compared to the non-TE control (Figure 6A). By dissociating the datasets in TE classes, we also observed global enrichments for each class in all TE regions compared to control TEs, especially for NR5A1 and GATA motifs (Supplementary Figure S4B). For other motifs, the enrichment is less obvious, sowing some specificity of classes.

Importantly, we also found sexual dimorphism between Sertoli and pre-granulosa in TEs bearing the same histone marks. Interestingly, motifs for male-specific transcription factors, such as SRY/SOX and DMRT1, were more represented in Sertoli-accessible TEs than in pre-granulosa cells independent of their associated histone marks (Figure 6D) with similar results when analyzing different classes (Supplementary Figure S4B). Likewise, pre-granulosa-specific transcriptions factors (FOXL2 and RUNX1) also displayed some sexual dimorphisms (Figure 6C and Supplementary Figure S4B), but to less extent than what we observed for the Sertoli-specific motifs. However, FOXL2 motifs present a different pattern when analyzed by classes; indeed, this motif has mostly depleted in SINEs of open TEs while being more present in other classes. Finally, motifs for transcription factors involved in the formation of the primordial gonads, and later on the maintenance of both Sertoli and pre-granulosa cells, displayed less sexual dimorphism such as GATA, WT1, and NR5A1, which were present at the same frequency in both Sertoli- and pre-granulosa cell-accessible TEs regardless of their classes (Figure 6B and Supplementary Figure S4B).

Our results demonstrate that accessible TEs in Sertoli and pre-granulosa cells are enriched in motifs recognized by the major transcription factors involved during gonadal differentiation. In addition, the motifs recognized by male transcription factors are globally more present in TEs opened in Sertoli cells than in pre-granulosa cells in all subsets of accessible TEs. As random TEs are not naturally enriched for gonadal transcription factor motifs, this suggest that these TEs in male and female supporting cells may have evolved to acquire *de novo* motifs recognized by transcription factors involved in the regulation of their respective transcriptional programs.

## Discussion

In this work, we analyzed, for the first time, the TE ecosystem in mouse developing gonads during the time window of sex determination. We observed that approximately 17,000 individual TE loci are transcribed independent of gene promoters and that their expression is tightly regulated in a sex- and time-specific manner as the bipotential gonads adopt their ovarian or testicular identity. Some of the differentially expressed TE loci are found in close proximity to protein-coding genes (100 kb) and their expression correlates with their proximal genes. At the chromatin level, we showed that TE loci are acquiring cell-type-specific enhancer- and promoter-like characteristics while the gonadal somatic cells specify as pre-granulosa or Sertoli cells in XX and XY gonads, respectively. DNA motif analysis revealed that TE loci are displaying potential binding sites for the critical gonadal development and sex-determining transcription factors. This study resulted in the identification of TE-derived regulatory elements that can participate and contribute to the complex gene regulatory network controlling gonadal development in mice.

The unbiased measurement of TE expression at a locus-specific level remains a challenge using shotgun sequencing technologies. Due to their repetitive nature, short reads cannot be easily attributed to a specific locus, unless TEs have accumulated mutations along evolution. As such, despite the use of EM algorithms that re-allocate multi-mapped reads, TE quantification is biased toward ancient TEs compared to recently inserted TEs displaying fewer mutations. The same challenge is even aggravated when analyzing genomic sequencing data, such as ATAC-seq and ChIP-seq, as no specific tool exists to attempt to re-allocate the multi-mapped reads. Hence, the current analysis is underestimating the number of individual TE loci that are transcribed or accessible in mouse gonads. The use of long-read sequencing technologies would allow unambiguously mapping individual TE loci with fragments longer than TEs and would result in a more resolutive overview of the TE landscape.

Our analyses are based on RNA sequencing of whole gonads and raised the question as to whether TEs might be expressed in a cell-type-specific manner. We first intended to reanalyze single-cell transcriptomic data (Stevant et al., 2018; Stevant, et al., 2019); however, such technologies revealed to not be sensitive enough to efficiently detect TE expression (10,000 total expressed TE loci in an average per stage, against 75,000 in the present bulk RNA-seq, data not shown). Using publicly available bulk RNA-seq data, we could not evaluate whether the expressed TEs we measured in the whole gonads were originating from somatic cells or germline compartments. We reanalyzed the datasets obtained at E11.5 from purified somatic precursor cells and showed that at this stage, half of the self-expressed TEs expressed in the whole gonads were commonly expressed in the purified somatic cells. Moreover, previous analysis at E13.5 (Liu, et al., 2014) suggests that some endogenous retroviruses are much more expressed in somatic cells than in the germ cells of fetal testes. Ultimately, the high-quality RNA-seq data of purified cell types composing the developing gonads of both sexes, similar to what has been done using microarray technology (Jameson, et al., 2012), would greatly contribute to the field to study unexplored aspects of the gonadal transcriptome, with a better sensitivity than the currently existing single-cell data.

An important question is what might be the role of these expressed TEs during gonadal development? In the early embryo, which is the most investigated model for TE expression to date, it has been shown that the activation of LINE-1 expression is required for global chromatin accessibility, independent of the coding nature of the transcript (Jachowicz, et al., 2017). While rarely present in protein-coding genes, co-opted TEs, particularly retrotransposons, constitute a source of lncRNAs (Kelley and Rinn, 2012; Kapusta, et al., 2013) known to be necessary for the correct execution of cellular processes. The endogenous MERVL, expressed in the two-cell stage (Svoboda et al., 2004), is a marker of totipotent cells (Macfarlan et al., 2012). The TE transcript, but not the encoded protein, is required for the correct development of the pre-implantation embryo, possibly through chromatin remodeling during the totipotent–pluripotent transition. At the mechanistic level, it has been shown that LINE-1 RNA recruits nucleonin and TRIM28 to regulate some target genes in embryonic stem cells (Percharde, et al., 2018). During neurogenesis, TE RNAs are associated with chromatin and regulate the activity of polycomb repressor complexes PRC2 (Mangoni et al., 2023). The dynamics of TE expression suggests that some TEs expressed uniquely at E10.5 could contribute to the multipotent state of the cells prior to sex determination, while TEs expressed during and after cell specification and in a sex-specific manner might be involved in the cell differentiation process. Targeted transcriptomic silencing or ablation of these TE loci using CRISPR technologies would allow to functionally explore their role and understand the mechanism of action.

Chromatin architecture influences gene transcription by modulating the access of *cis*-regulatory elements to transcription factors. In this study, we identified that half of the DNA regions that gain accessibility while the gonadal progenitor cells differentiate as pre-granulosa and Sertoli cells overlap with TE loci. A large proportion of these TEs are displaying distinctive histone marks for enhancers and promoters (H3K4me3 and H3K27ac). Using a similar approach, previous studies have identified lineage-specific TE-derived *cis*-regulatory elements (Fueyo, et al., 2022). However, a functional evaluation survey in mouse embryonic and trophoblast stem cells using genome-editing technologies revealed that few of them are critical for gene regulatory networks (Todd et al., 2019). We predict that most candidate TE-derived cis-regulatory regions identified in this study are not critical players of supporting cell differentiation, but rather redundant or shadow enhancers that help sustain the gene regulatory network. However, we cannot exclude that specific TE loci may have become the key players of the gonadal sex determination process. The presence of TEs within TESCO suggests their role in its enhancer activity. The TESCO sequence is highly conserved across mammals (Bagheri-Fam et al., 2010). In mice, TESCO contains four different TE sequences [one DNA TcMar-Tigger and three LINEs, including a B4 and two mammalian-wide interspersed regions (MIRs)]. These four TEs are conserved in rats, while only two LINEs from the MIR family are found across multiple mammalian species, such as rat, rabbit, human, and tree shrew (cf. https://genome.ucsc.edu). These TEs contain predicted binding motifs for the SOX and GATA family of transcription factors (cf. https://genome.ucsc.edu). As such, they potentially contribute to the specie-specific characteristics of TESCO activity. In the same line, several studies have shown that TEs are highly heterogeneous between mouse strains (Nellaker et al., 2012; Jung et al., 2023) that can modify gene expression (Zhou et al., 2021) and chromatin dynamics (Ferraj et al., 2023). Therefore, we can speculate that strain-specific TEs could be involved in the sensitivity of a C57BL/6J background to sex reversal (Eicher et al., 1982; Eicher et al., 1996; Munger et al., 2009).

The enrichment of critical gonadal transcription factor-binding motifs in these accessible TE loci in the differentiating supporting cells further substantiates that TEs could have been co-opted as *cis*-regulatory elements in the context of gonadal sex determination. The enrichment of TE sequences in NR5A1-binding motifs, regardless of their chromatin landscapes, shows that this motif is seemingly already present in the ancestral TE sequences and will be even more enriched in the accessible TEs of both sexes. In contrast, the specific enrichment of gonadal transcription factor motifs in the accessible TE loci in a time- or sex-specific manner could be explained by *de novo* motif acquisition and/or by a co-optation of a particular TE subfamily that naturally displays these motifs (Garcia-Perez et al., 2016). To support this, our results show a clear enrichment for male-specific transcription factors (SRY/SOX and DMRT1) for the TEs specifically accessible in Sertoli cells compared to pre-granulosa cells. Our refine study of motif enrichment in TE classes shows a global conservation of the enrichment for SRY/SOX and DMRT1. It is also the case for TF motifs involved in the formation of early gonads such as NR5A1, GATA, and WT1; for the same sets of TEs, motifs are mostly enriched in the same manner when they are analyzed by class. One exception is FOXL2 motifs, linked to pre-granulosa cell differentiation, where we observed significant differences between the different classes analyzed. Concerning strain specificity, the motif composition might vary depending of the mouse strain and influence subsequent recognition by DNA-binding proteins as it has been shown for CTCF in the mouse CD-1 strain (Jung, et al., 2023).

In light of our findings, suggesting that TEs may participate in sex determination, it will be interesting to reanalyze human patients suffering from difference in sexual development (DSD) with unexplained etiology (more than 50%: (Rakover et al., 2021)) to investigate whether the reshuffle of TE loci is the cause for their phenotypes. Indeed, the development of long-read technologies of sequencing and dedicated bioinformatics tools for analysis of repeated sequenced would allow better resolving these questions.

## Materials and methods

### RNA-seq mapping and quantification

RNA-seq FastQ files from the *Trim28* knockout mouse ovaries (Rossitto, et al., 2022) (GSE166385), the *Dmrt1* ovarian reprogramming (Lindeman, et al., 2015) (GSE64960), the whole embryonic gonad splicing event (Zhao, et al., 2018) (SRP076584), and Nr5a1-GFP+ purified somatic cells at E11.5 (Miyawaki, et al., 2020) (GSE151474) studies were downloaded from Gene Expression Omnibus using the nf-core/fetchngs pipeline v1.10.0 (Ewels et al., 2020; Patel et al., 2023a).

Gene- and locus-specific TE expressions were measured using SQuIRE v0.9.9.92 (Yang, et al., 2019). In brief, FastQ files were mapped with STAR v2.5.3a on the 10-mm mouse reference genome obtained from the UCSC. Gene and TE quantification were performed using the 10-mm gene annotation and RepeatMasker TE annotation from the UCSC. First, uniquely mapped reads were assigned to their corresponding TE loci. Then, the multi-mapped reads were assigned to TE loci using an EM algorithm. A score was calculated for each TE locus to account for the proportion of reads uniquely mapped and re-attributed multi-mapped reads in the total TE read count. As TEs with few uniquely aligning reads and numerous re-attributed multi-mapped reads may be prone to low confidence quantification, we considered TEs displaying a score >95 and a minimum of five reads covering the TE locus. We also excluded TEs located outside the conventional chromosomes. Subsequent analysis was performed with R version 4.2.2 “Innocent and Trusting.” TE counts together with gene counts were normalized by library size using DESeq2 prior to analysis (Love et al., 2014).

### Expressed TE classification and annotation

TEs were classified as gene-dependent or self-expressed if they were found within expressed genes or in intergenic regions or non-expressed genes. To proceed, SQuIRE read counts for genes and TEs were loaded in R. TE loci and 10-mm gene annotation GTF files were transformed as genomic range objects using the GenomicRanges (Lawrence et al., 2013) and the rtracklayer packages, respectively (Lawrence et al., 2009). TE and gene overlaps were computed using “GenomicRanges::findOverlaps” with the option “ignore.strand = TRUE.” For each sex and embryonic stage, we investigated whether the genes overlapped by TEs are expressed with a minimum of 10 reads per gene. TEs located within an expressed gene were classified as gene-dependent, as we consider their expression driven by the gene promoter, and the rest of the TEs were classified as self-expressed.

The annotation of TEs as intergenic, exonic, or intronic was performed using “ChIPseeker::annotatePeak” (Wang et al., 2022) using the same 10-mm GTF file as the TE quantification with SQuIRE for genome annotation version consistency.

### TE family enrichment test

The TE family enrichment test was performed using the “fisher.test” R function as described by Chang, et al. (2022). For each TE family, we created a contingency table with the number of expressed TEs that belong or do not belong to the family, as well as all annotated TEs that belong or do not belong to the family, and we used Fisher’s exact test. *p*-values were corrected for a false discovery rate using the “p.adjust” function with the Benjamini–Hochberg method.

### TE differential expression analysis

Differential TE expression analysis by embryonic stage and sex was performed using DESeq2 using both TE and gene quantification for a correct normalization of the data. First, a sample sheet was built with the “stage_sex” sample annotation (e.g., “E10.5_XX”) for each sample. A DESeq2 object was created using “DESeqDataSetFromMatrix” with “∼stage_sex” as design.

Sexually dimorphic TEs were obtained with the “DESeq” function with default parameters (Wald test). XX and XY overexpressed genes per stage were recovered using the “results” function with “stage_XX” and “stage_XY” contrast for each stage. Dynamically expressed TEs computed sex independently. For each sex, dynamic TEs were obtained using the “DESeq” function with test = “LRT,” reduced = ∼1′ options (likelihood-ratio test). In both differential expression analyses, we considered a TE statistically differentially expressed with an adj.pval<0.05.

Dynamically expressed TEs per sex were then represented as a heatmap with ComplexHeatmap (Gu et al., 2016) with rows normalized with z-scores and classified into seven clusters using the “Ward.D2” clustering method. The number of clusters was chosen by visual inspection of the heatmap split.

### TE-gene correlation

To inspect whether TE expression was correlating with nearby gene expression, we calculated TE-gene expression correlation per sex across different embryonic stages. To identify the genes located near the time and sex differentially expressed TEs, we first checked the average distance of TE and the nearest gene TSS. We observed that most TEs are located up to 100 kb away from a gene TSS (Supplementary Figure S2D). We defined a window of 100 kb upstream and downstream of the time and sex differentially expressed TEs and selected all the genes overlapping these windows. Then, we calculated Pearson’s correlation between TEs and their nearby gene expression across all embryonic stages. We considered a positive correlation with *ρ* > 0.6 and *p*-value<0.05 and a negative correlation with *ρ* < −0.6 and *p*-value<0.05.

To visualize the expression correlation between TEs and their nearby genes, we used the bigWig files generated with SQuIRE with the “draw” script. In order to have a clean representation of TE expression and avoid seeing other nearby TEs that were previously excluded by our quality filters described above, we used the bigWig files containing the uniquely mapped reads only and not the re-attributed reads. The genomic track visualization was built using the Gviz R package (Hahne and Ivanek, 2016).

### ATAC-seq mapping and analysis

FastQ files for the ATAC-seq data from E10.5 and E13.5 early gonadal progenitors and supporting cells, respectively (Garcia-Moreno, Futtner, et al., 2019), were downloaded from Gene Expression Omnibus (GSE118755) using the nf-core/fetchngs pipeline v1.10.0. Data were mapped on the 10-mm reference genome and analyzed using the nf-core/atacseq v2.1.2 (Patel et al., 2023b). Sequencing data quality was assessed using FastQC, sequencing adapters were removed with cutadapt, and reads were mapped using BWA. Reads were filtered with Picard to remove unmapped and duplicated reads. Peaks were called with MACS2 using the “narrow peak” parameter. For the subsequent analysis, we selected the peaks found in at least two replicates.

Differential accessibility analysis between XX and XY for each stage as well as E10.5 and E13.5 for each sex was performed using DESeq2. First, a consensus table of the read counts per accessible regions was built with featureCounts using the nf-core/atacseq pipeline. Time-specific and sexually dimorphic accessible regions were obtained with the “DESeq” function with default parameters. Sex-biased accessible regions per stage and stage-biased accessible regions per sex were recovered using the “results” function with “stage_XX’ and “stage_XY” contrast for each stage and “E10.5_sex” and “E13.5_sex,” respectively. A region was defined as differentially accessible with an adj.pval<0.05 and |log2 (foldChange)|>1.

TE overlapping the differentially accessible regions were recovered using “GenomicRanges::findOverlaps” with the option “ignore.strand = TRUE.”

### ChIP-seq mapping and analysis

FastQ files for the ChIP-seq data (H3K4me3, H3K27ac, and H3K27me3) from E10.5 and E13.5 early gonadal progenitors and supporting cells, respectively (Garcia-Moreno, Futtner, et al., 2019), were downloaded from Gene Expression Omnibus (GSE118755 and GSE130749) using the nf-core/fetchngs pipeline v1.10.0. Data were mapped on the 10-mm reference genome and analyzed using the nf-core/chipseq v2.0.0 (Ewels et al., 2022). Similar to the ATAC-seq analysis, sequencing data quality was assessed using FastQC, sequencing adapters were removed with cutadapt, and reads were mapped using BWA. Reads were filtered with Picard to remove unmapped and duplicated reads. Peaks were called with MACS2 with the respective input controls using the “narrow peak” parameter for H3K4me3 and “broad peaks” for H3K27ac and H3K27me3. Because of the differences of sequencing depth, mapped reads, and peak numbers between the replicates, we considered all the peaks called from any replicates for the rest of the analysis.

### TE loci epigenetic classification

We first selected TEs that contributes to the differentiation of the supporting cells as either Sertoli or pre-granulosa cells. To proceed, for each sex, we selected the TE displaying an accessible chromatin region that is statistically more accessible at E13.5 than E10.5, as well as the accessible chromatin regions that are sexually dimorphic at E13.5. We extended the coordinates of the obtained TEs by 200 bp to look for ChIP-seq peaks in the direct vicinity of the TEs, at a one nucleosome resolution. We overlapped the obtained TE regions with the ChIP-seq peaks from E13.5 H3K4me3, H3K27ac, and H3K27me3 for both sexes. For each TE, we binarized the presence of either H3K4me3, H3K27ac, or H3K27me3, i.e., we marked 1 if one or more peaks were found or 0 if no peak was found, and checked the presence of the following combination of histone marks that are biologically relevant: only H3K4me3 (promoters), H3K4me3 and H3K27ac (promoters), only H3K27ac (active enhancer), only H3K27me3 (silencer), and H3K4me3 and H3K27me3 (poised promoters). The other possible combinations were labeled as “Others,” and the loci overlapping none of these histone marks were labeled as “No peak.” The histone marks overlapping the open TEs were represented on heatmaps with EnrichedHeatmap (Gu et al., 2018) using merged replicate bigWig files produced with WiggleTools and bedGraphToBigWig utilities. For visualization clarity, the combinations of histone marks with fewer than 500 open TEs were grouped in the “Other” section as the groups were not clearly visible on the final heatmap.

### GO-term enrichment analysis

Biological process GO-term enrichment analysis was performed using the g:profiler website (https://biit.cs.ut.ee/gprofiler/gost) using the mouse genome as the background, Benjamini–Hogberg FDR <0.5. Because of the large amount of genes present in different analyses, GO terms were displaying various general processes, such as “Developmental processes.” For the concision of the reported results, we filtered the obtained statistically enriched GO terms to maintain biologically relevant terms containing not only “sex,” “reproduction/reproductive,” and “gonad” but also “Wnt” in female-related gene lists and “angiogenesis” and “epithelium” for male-related gene lists.

### TE conservation

The conservation of mouse (10 mm) TE sequences between rat (rn7), human (Hg38), cow (bosTau9), and chicken (galGal6) was performed using LiftOver from the UCSC website (https://genome.ucsc.edu/cgi-bin/hgLiftOver) with default parameters. As a reference, we selected 50,000 random TE sequences and checked which percentage was found conserved in all the species mentioned above. We then repeated the same procedure with all groups of sex- and time-specific accessible TEs carrying different combinations of histone marks in pre-granulosa and Sertoli cells.

### TF motif enrichment analysis

Binding sites for transcription factors involved in mammalian sex determination/differentiation were counted using the matrix scan (full options) algorithm from the RSAT suite (http://www.rsat.eu) (Santana-Garcia et al., 2022). The DNA-binding preferences for the transcription factor analyzed were modeled as matrices and analyzed with default settings and a *p*-value set to 10^−4^. Matrices for transcription factors were obtained from the JASPAR database (https://jaspar.genereg.net): SRY (MA0084.1); SOX9 (MA0077.1); SOX8 (MA0868.2); SOX10 (MA0442.2); SF1, also known as NR5A1 (MA1540.1); GATA4 (MA0482.1); GATA6 (MA1104.1); DMRT1 (MA1603.1); FOXL2 (MA1607.1); RUNX1 (MA0002.2); and WT1 (MA1627.1). GATA4 and GATA6 are redundant during gonadal differentiation (Padua, et al., 2014; Padua, et al., 2015), and the HMG domain of SOX proteins displays related functions (Bergstrom et al., 2000; Polanco et al., 2010). Therefore, the pooled results of matrices scanning for GATA4 and GATA6 were expressed as “GATAs” and “SRY/SOX” for pooled scanning with SRY, SOX8/SOX9/SOX10 matrices. To normalize counts, each motif was expressed in the number of matches per kilobases of the total length of the datasets. Statistical analyses were made using GraphPad Prism 10 with ordinary one-way ANOVA and Tukey’s multiple comparison test. The detailed results of statistical tests are provided in Supplementary Table (Supplementary Data S6). *De novo* motif analyses were performed using peak-motifs from the RSAT suite.

## Data Availability

The original contributions presented in the study are included in the article/Supplementary Material; further inquiries can be directed to the corresponding authors.

## Supplementary material

Supplementary Figure 1 Differential TE expression analysis of two cases of granulosa-to-Sertoli transdifferentiation in adult ovaries. **(A, B)** Heatmap representing the differentially expressed TEs in the *Trim28* knock-out ovaries **(A)** and the *Dmrt1* over-expression ovaries **(B)** models compared to control ovaries, respectively. Expression data were normalized using z-scores. **(C)** Overlap of the differentially expressed TEs in the *Trim28* knock-out and the *Dmrt1* over-expression models. Despite the differences in mouse strain genetic background, RNA-seq library, and sequencing protocols, 655 and 251 TEs were found commonly up and down regulated after granulosa-to-Sertoli transdifferentiation.

Supplementary Figure 2 Characteristics of the TE found expressed in mouse fetal gonads. **(A)** Number of TE loci detected as expressed across sexes and stages. **(B)** Comparison of the self-expressed TEs detected in whole gonads (Zhao’s dataset) and purified somatic cells (Miyawaki’s dataset) at E11.5 in XX and XY gonads. Due to the difference in the RNA-seq library preparation protocols and sequencing sensitivity, more self-expressed are detected in the Miyawaki’s dataset. However, half of the Zhao’s self-expressed TEs were also found in Miyawaki’s dataset. **(C)** Proportion of the self-expressed TE families across sexes and stages. **(D)** Distance of the sex- or time-differentially expressed self-expressed TEs relatively to the nearest gene TSS. The red square shows a magnification of the TSS region, +/- 100 kb where most of the TEs were found.

Supplementary Figure 3 Characteristics of the ATAC-seq peaks overlapping TEs. Number of TEs contained within ATAC-seq peaks (green) and percentage of the TE sequences overlapping with ATAC-seq peaks (salmon) are reported for each sex and stage. The ATAC-seq peaks can contain up to eight TEs but most of them contains one or two individual TE loci. Most of the TE sequences are entirely embedded within the ATAC-seq peak (100%).

Supplementary Figure 4 Transcription factor binding motif enrichment in pre-granulosa and Sertoli cell per classes of accessible TE loci. **(A)** Ratio of the number of the respective transcription factor motifs found in 3 sets of 10,000 randomly picked TE dissociated in four different classes (LINEs, LTRs, SINEs and others) or non-TE sequences, respectively. Motifs were analyzed and normalized as described in Figure 6A. **(B)** Same analyses as in Figure 6B-D for the different set of accessible TEs dissociated in four different classes (LINEs, LTRs, SINEs and others) and reported to corresponding classes of three different sets of 10,000 control TEs. XY and XX correspond to accessible TEs from Sertoli and pre-granulosa cells respectively.

Supplementary Figure 5 Expression level of the gonad transcription factors. Expression of the transcription factors used for the motif enrichment analysis across fetal gonad development. Data are expressed as TPM (transcript per million).

Supplementary Table 1
*De novo* motifs analysis for each set of the accessible TEs from Sertoli (XY) and pre-granulosa cells (XX). Are reported all transcription factors motifs others than those already used in Figure 6.

Supplementary Datasheet 1 Temporally differentially expressed self-expressed TEs. Results of the differential expression analysis across embryonic stages for XX and XY gonads using DESeq2. Only results with padj < 0.05 are reported. The column “TE name” refers to the TE locus identifier which is represented as chromosome|start|end|TEsubfamily:TEfamily:TEclass|size|strand. The “baseMean” is the mean of normalized counts for all samples. “pvalue” column is the *p*-values resulted from the LRT test, and “padj” is the *p*-value corrected for false discovery rate using Benjamini-Hochberg. The column “cluster” refers to the TE clustering presented on the heatmaps from Figure 2A. XX and XY results are reported in separated tabs of the spreadsheet.

Supplementary Datasheet 2 Sexually differentially expressed self-expressed TEs. Results of the differential expression analysis between XX and XY gonads for each embryonic stages using DESeq2. Only results with padj < 0.05 are reported. Similarly to Sup. Data 1, the column “name” refers to the TE locus identifiers, the “baseMean” is the mean of normalized counts for all samples, “log2FoldChange” of the fold change in expression of XX vs XY normalized expression conditions transformed with log2. Positive values indicate over-expression in XX, and conversely, negative values indicate overexpression in XY. “lfcSE” columns gives the standard error of the log2FoldChange. “pvalue” is the *p*-values resulted from the WALD test, and “padj” is the *p*-value corrected for false discovery rate using Benjamini-Hochberg. The columns “sex” and “stage” refer to the sex and stage in which the TE is overexpressed.

Supplementary Datasheet 3 Sex- and time-differentially expressed TE correlation with genes in their 100 kb neighborhood. Results of the TE-gene correlation analysis. “TE” column refers to the TE locus identifiers, the “Gene” column are the symbol of the genes present in the 100 kb regions around the TEs. The distance of the gene TSS to the TE is referred in the “distance_to_gene” column. XX and XY Pearson correlation coefficients and their respective p-values are referred. Mathematically impossible correlation test (*i.e.* when the gene expression value is equal to 0) were annotated with a correlation coefficient of 0 and a *p*-value noted “NA”. The “TE_coord” column gives the TE loci coordinates in a format usable in IGV genome browser for convenience.

Supplementary Datasheet 4 Classification of the TEs gaining accessibility in a sex- or time-specific manner in pre-granulosa and Sertoli cells according to histone marks. The spreadsheet presents three different tabs. The first tab contains the number of accessible TEs for each different biologically relevant histone mark classification. The second and third tabs contain the classification details for the pre-granulosa and Sertoli accessible TEs respectively. The first five columns refer to the TE coordinates and their identifier, columns six to eight refers to the presence or not of the respective histone marks as binary data (*i.e.* “0” if no peak was found in the ChIP-seq data, “1” if one or more peaks were found). The “category” column refers to the classification of the TE according to the histone marks found in their sequence. “annotation” are the genomic locations of the TEs, *e.g.* exonic, intronic or intergenic. “geneid” and “distanceToTSS” refer to the nearest gene TSS and the distance from the TE loci.

Supplementary Datasheet 5 GO-term enrichment for the proximal genes to the sex- and time-accessible TEs found in pre-granulosa and Sertoli cells according to histone marks. The spreadsheet presents two tabs. The first shows the results for the pre-granulosa accessible TEs, while the second the results for the Sertoli cell. GO-term enrichment results were filtered to show biologically relevant terms.

Supplementary Datasheet 6 Details of the statistical tests used to compare the enrichments of motifs between the different sets of open TE and control TE. The spreadsheet presents two tabs. The first shows the summary of all statistical tests and the seconds the details of each test. *P* < 0.032 (*), < 0.0021 (**), < 0.0002 (***), < 0.0001 (****). The performed was ordinary one-way ANOVA. Multiple comparisons with no matching and pairing was performed using Tukey’s test.
