# Supplementary material for: Transposable elements acquire time- and sex-specific transcriptional and epigenetic signatures along mouse fetal gonad development
Source: Front Cell Dev Biol. 2024 Jan 12;11:1327410. doi: 10.3389/fcell.2023.1327410 (PMC10811072; doi:10.3389/fcell.2023.1327410)
Supplement: Supplementary file 1 [file Table1.docx]

| Open TEs | Sex | De novo motif analyses using Peaks-motifs (RSAT) and JASPAR database |
| --- | --- | --- |
| H3K27ac | XY | NR5A2, NR6A1, NR4A1, ESRRG, ESRRA, Nr2e3, Ahr::Arnt, HAND2, OSR2, ETV4, ETV5, ZNF460, STAT5B, STAT3, GFI1b, PRDM4, PRDM1, SMAD2::SMAD3::SMAD4, TFAP2A, TFAP2B, SNAI1, OTX2, PITX3, ZIC1::ZIC2, FIGLA, |
|  | XX | NR5A2, NR6A1, ESRRA, ZNF384, PRDM1, STAT2, ZNF384, VDR, ZNF384, MEF2A, CDX2, ZNF384, FOXP1, ASCL1(var.2), SNAI1, EHF, ZNF384, STAT2, STAT1::STAT2. |
| H3K4me3 | XY | NR2E3, ESRRA, ESRRB, TBP, HOXD9, HOXA10, CDX4, MSANTD3, NR1I3, VDR, MNX1, EVX1, EVX2, FOXB1, FOXL1, |
|  | XX | NR6A1, ESRRA, NR5A2, ESRRB, ZNF384, EHF, ASCL1(var.2), SNAI2, ZNF384, ZNF148, OXL1, NFIL3, NKX3-1, OTX2, OTX1, PITX2, MAZ, KLF4, PRDM4, PRDM1, MEIS1, |
| H3K27ac + H3K4m3 | XY | NR5A2, NR2F2, NR4A2, NR6A1, NR2E3, ESRRA, ESRRB, AHR::ARNT, ZBTB12, ZBTB6, ETV4, ZNF460, TFAP2B(var.2), ZNF75D, RBPJ, KLF1, KLF2, KLF6, NR4A2, TFAP2A, TFAP2B(var.2), |
|  | XX | NR5A2, NR6A1, ESRRA, ZNF384, NFIL3, FOXD3, FOXL1, FOXC1, EHF, ASCL1(var.2), SNAI2, SNAI1, PRDM1, |
| H3K27m3 | XY | EAD4, RBPJ, TEAD1, KLF5, KLF4, KLF15, SP9, NFIX, ETV4, ETV6, ETV5, HOXB13, HOXD9, HOXA10, RFX1, RFX7, DMRTC2, CREB3L4, ZEB1, ZNF135, ZNF148, ESRRG, ESRRA, MAZ, RBPJ, |
|  | XX | ESRRA, PRDM1, STAT2, HOXA13, HOXD13, HOXB13, FOXL1, NFIL3, TEF, ZNF384, ZNF317, ZNF148, MAZ, KLF4, PHOX2A, PROP1, PHOX2B, |
| none | XY | NR5A2, NR4A1, NR4A2, ESRRA, ESRRB, ESRRG, TCF7, GFI1B, GFI1, BHLHE22(var.2), TCF12, MYOG, ETV4, ZNF460, ESRRA, NFIX, ZNF384, FOXQ1, FOXA1, MEF2D, MEF2B, HAND1::TCF3, RORA, PRDM15, TFAP2A, TFAP2B(var.2) |
|  | XX | NR5A2, NR6A1, NR4A2, ESRRA, ESRRB, RXRA, NR2E3, AHR::ARNT, ZNF384, ZNF460, ZNF75D, ZNF384, STAT2, E2F6, RBPJ, FOXJ3, FOXJ2, FOXQ1, EHF, TFAP2C(var.2), TFAP2B(var.2), STAT5B, STAT3, RBPJ |
