# Supplementary figures and images for: Transposable elements acquire time- and sex-specific transcriptional and epigenetic signatures along mouse fetal gonad development
Source: Front Cell Dev Biol. 2024 Jan 12;11:1327410. doi: 10.3389/fcell.2023.1327410 (PMC10811072; doi:10.3389/fcell.2023.1327410)

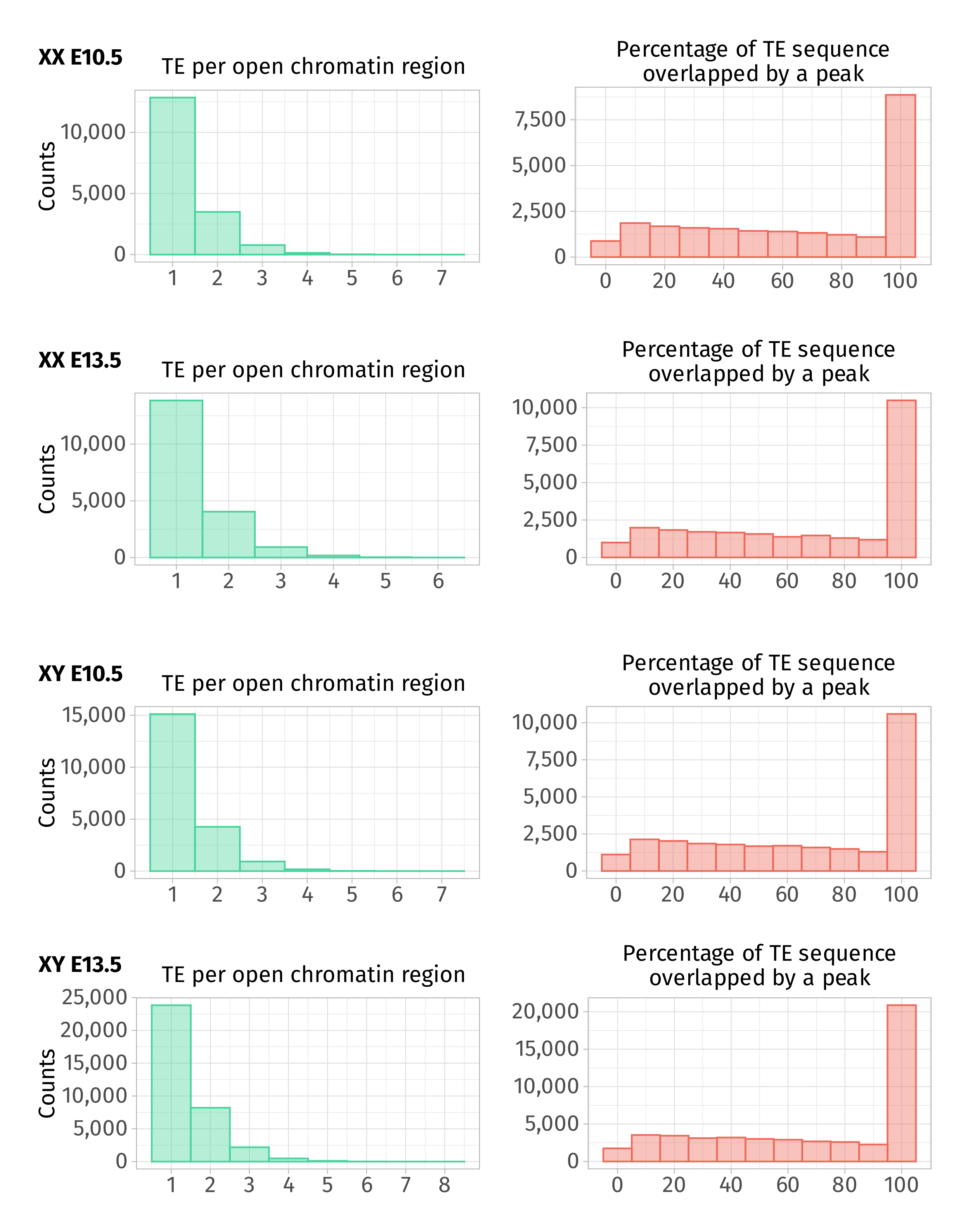

Supplement: Supplementary file 3 [file Image3.TIF]

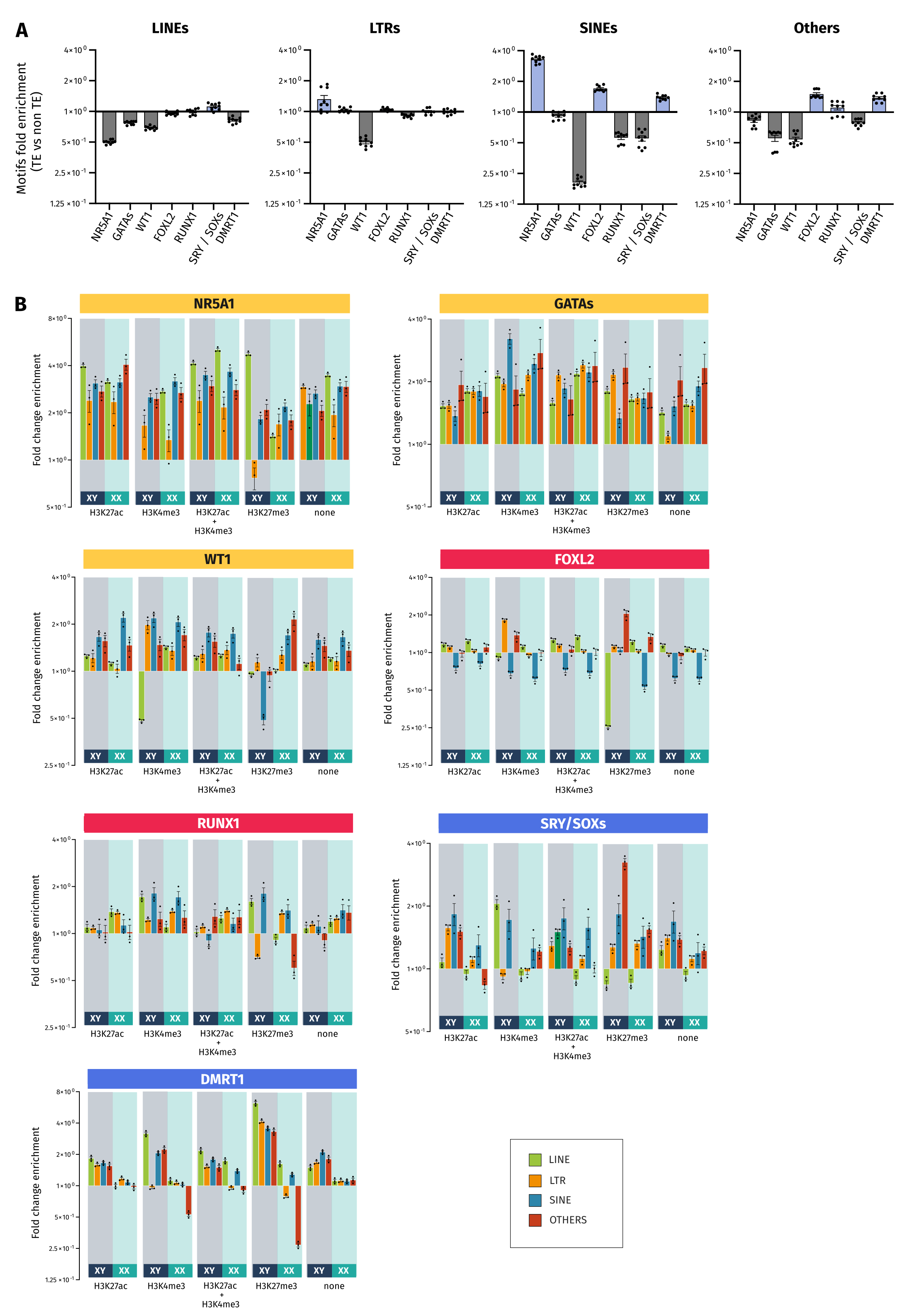

Supplement: Supplementary file 5 [file Image4.TIF]

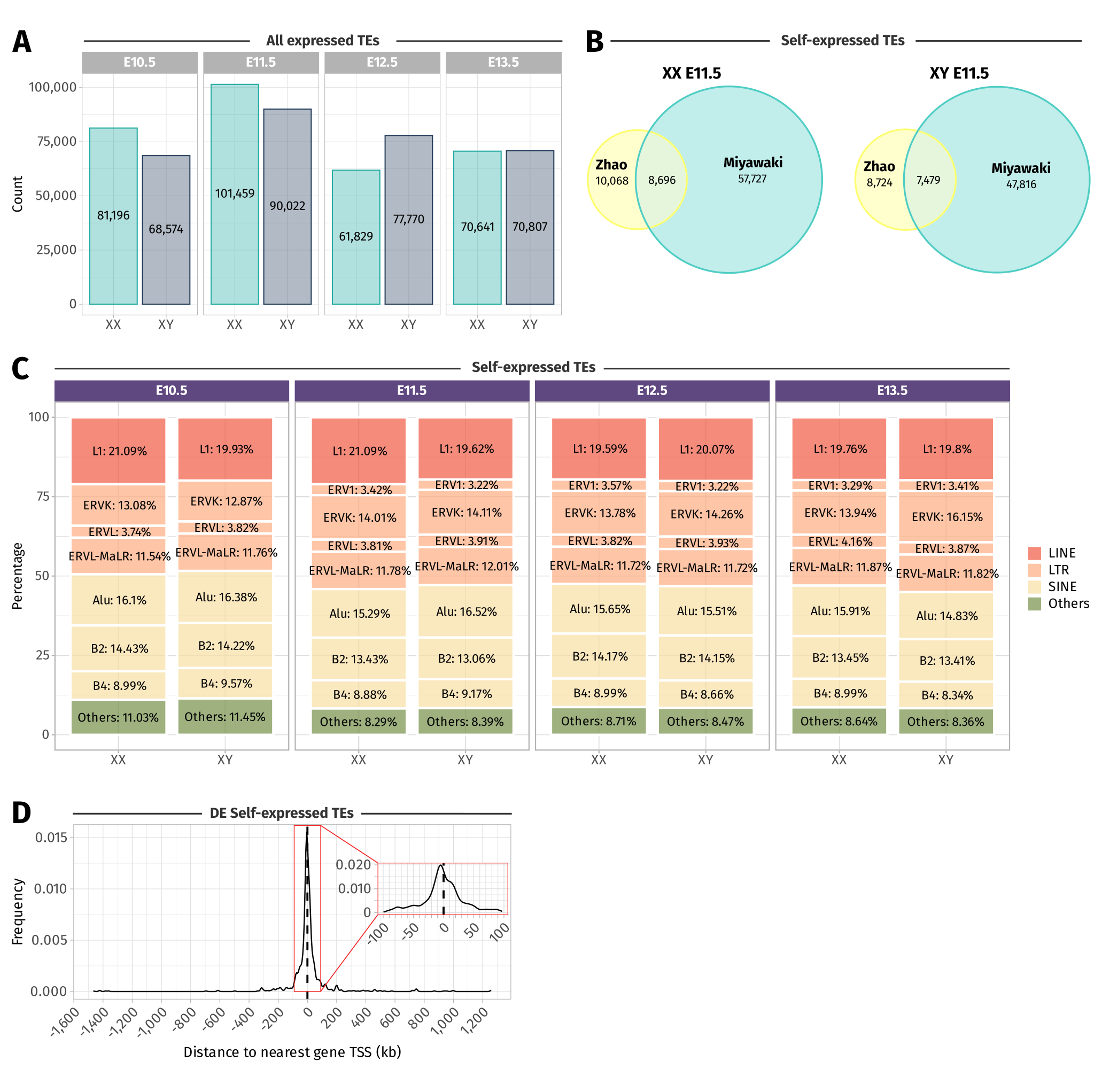

Supplement: Supplementary file 6 [file Image2.TIF]

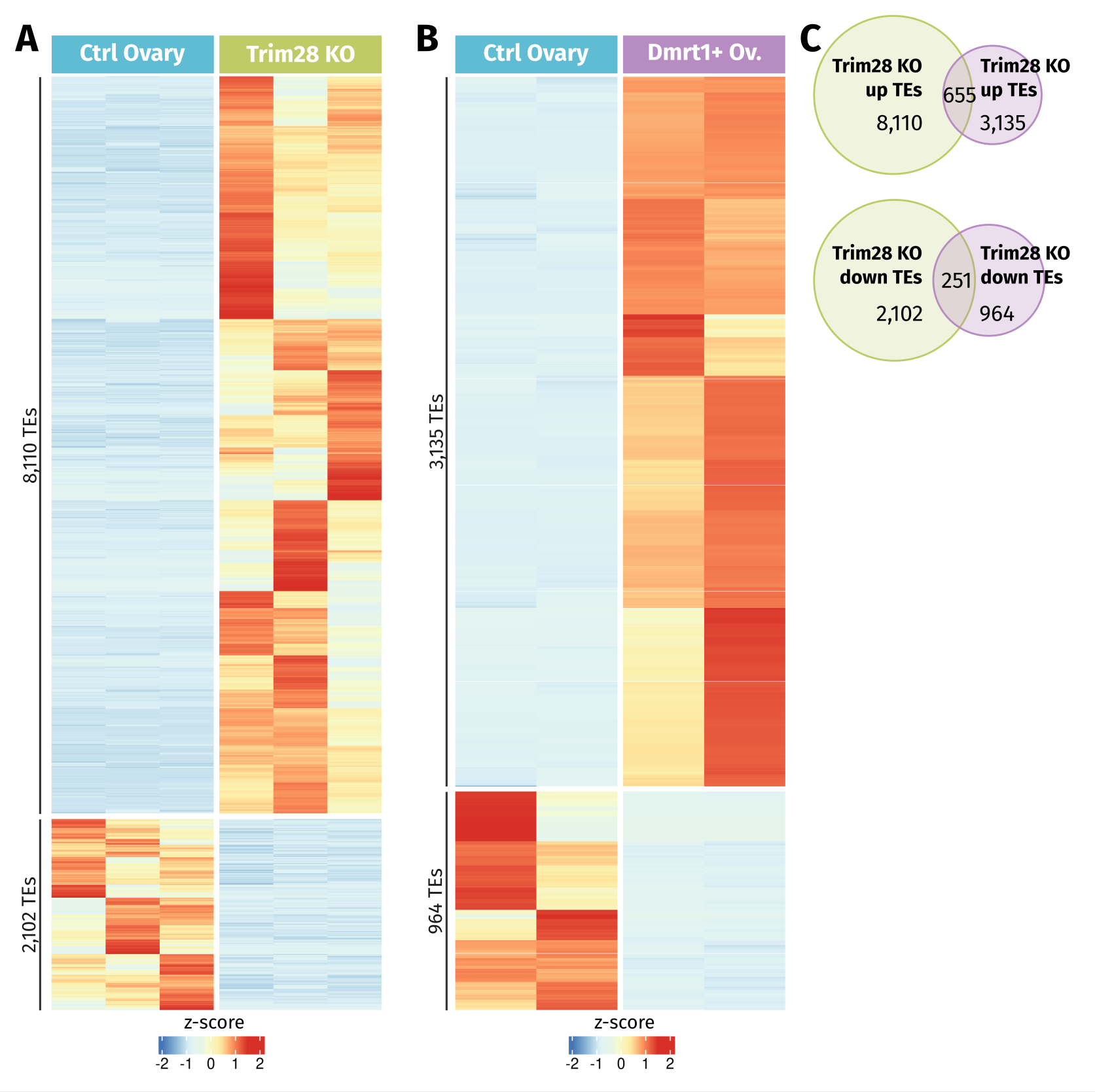

Supplement: Supplementary file 7 [file Image1.TIF]

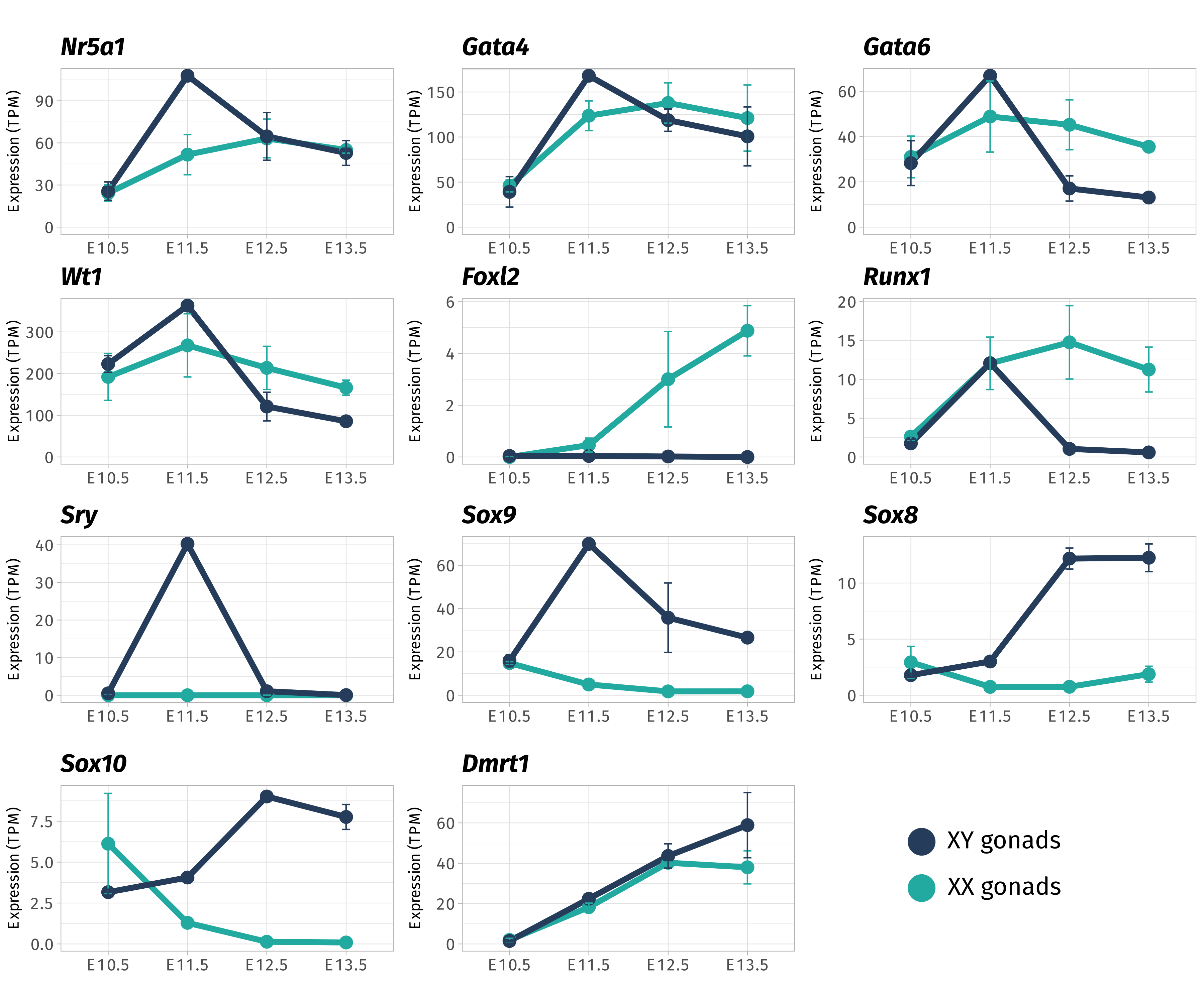

Supplement: Supplementary file 10 [file Image5.TIF]
